# 14-3-3/Tau molecular glues modulate *in vitro* tau condensation

**DOI:** 10.1039/d6cb00156d

**Published:** 2026-08-28

**Authors:** Maxime C. M. van den Oetelaar, Leandre Ravatt, Francisco Maqueda Zelaya, Ansgar Oberheide, Tom R. van de Wijngaart, Floor M. A. van Boxtel, Marloes A. M. Pennings, Carlo J. A. Verhoef, Thijs W. van Veldhuisen, Peter J. Cossar, Christian Ottmann, Susanne Wegmann, Luc Brunsveld

**Affiliations:** a Laboratory of Chemical Biology, Department of Biomedical Engineering and Institute for Complex Molecular Systems, Eindhoven University of Technology Groene Loper 3 5612 AE Eindhoven The Netherlands c.ottmann@tue.nl l.brunsveld@tue.nl; b Ambagon Therapeutics, Catalyst Business Center, De Lismortel 31 5612 AR Eindhoven The Netherlands; c Departamento de Química Orgánica y Química Inorgánica, Instituto de Investigación Química “Andrés M. del Río” (IQAR), Universidad de Alcalá, IRYCIS, Alcalá de Henares 28805 Madrid Spain; d German Center for Neurodegenerative Diseases (DZNE) Department Charité University Medicine, Charitéplatz 1 10117 Berlin Germany susanne.wegmann@dzne.de

## Abstract

The intrinsically disordered protein Tau is highly phosphorylated under pathological conditions, which, among others, results in Tau liquid–liquid phase separation (LLPS), followed by aggregation. The hub protein 14-3-3 regulates Tau protein solubility and prevents LLPS by binding with phosphorylated Tau residues pS214 and pS324. Here, we report the stabilization of this Tau/14-3-3 protein–protein interaction (PPI) using reversible-covalent small-molecule molecular glues. By strengthening the 14-3-3/Tau interaction the molecular glues enhance the effect of 14-3-3 on Tau solubility in LLPS. This demonstrates the potential of modulating the 14-3-3/Tau PPI for novel drug discovery efforts in neurodegenerative diseases.

## Abstract 2

Dynamic-covalent molecular glues stabilize the interaction between the 14-3-3 protein and two phosphorylated sequences of the Tau protein, thereby enhancing the solubilizing effect of 14-3-3 and inhibiting Tau liquid–liquid phase separation.

## Graphical abstract

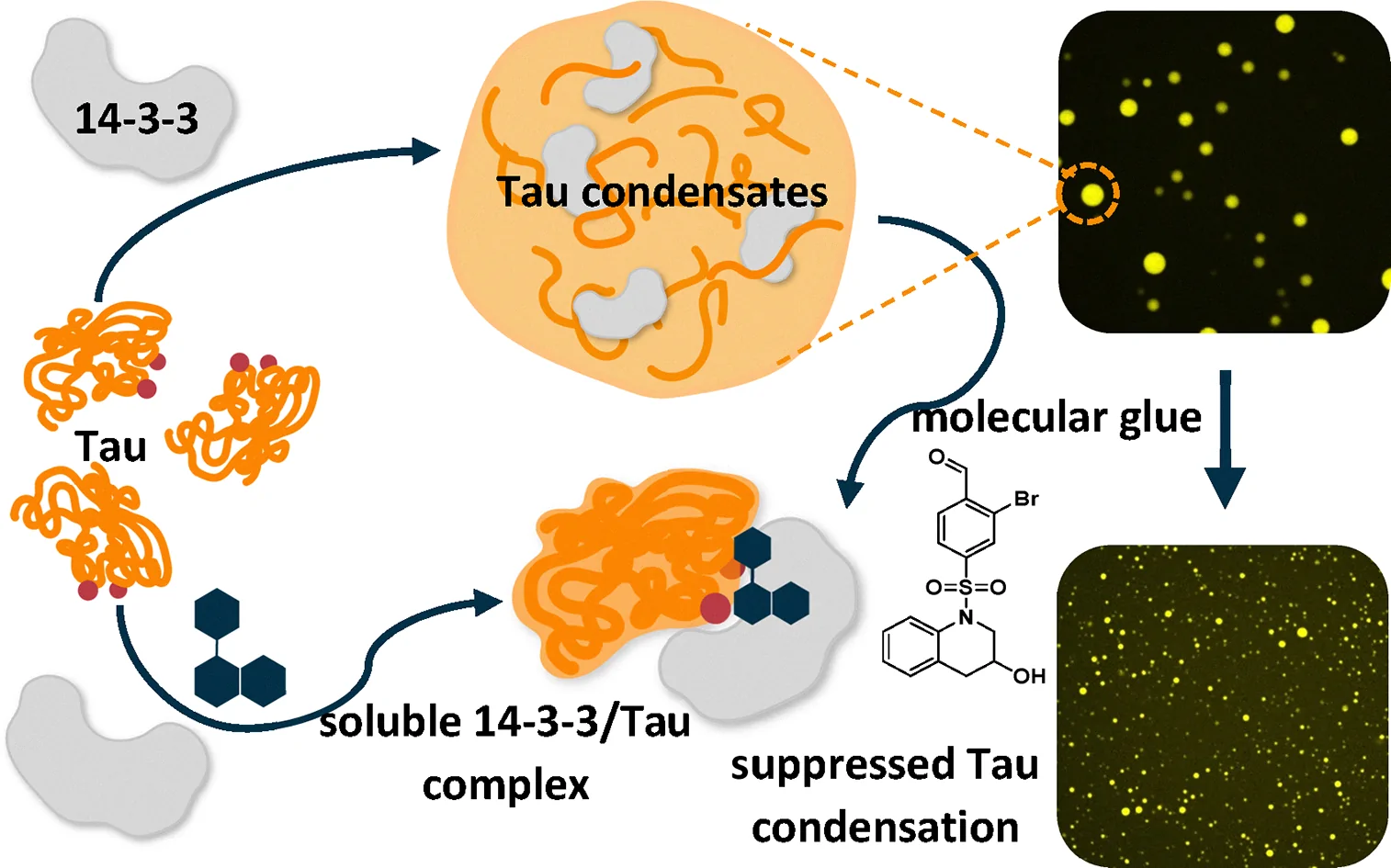

## Introduction

Tau is an intrinsically disordered protein (IDP) that stabilizes microtubules in the neuronal axon.^1–4^ Tau has over 80 putative phosphorylation sites with diverse biological roles.^3,5^ Under normal physiological conditions, Tau is typically phosphorylated at a limited number of sites, which regulate, among others, its microtubule-binding affinity as part of normal microtubule dynamics. Under pathological conditions, Tau becomes highly phosphorylated, resulting in excessive release of Tau from microtubules and, consequently, microtubule destabilization. This cytosolic accumulation of unbound phospho-Tau can promote the formation of toxic Tau aggregates.^1,6^*En route* to this, Tau can undergo liquid–liquid phase separation (LLPS).^4,7–10^

The hub-protein 14-3-3 has major regulatory roles in the human body by interacting with hundreds of partner proteins.^11–16^*Via* complex protein–protein interaction (PPI) networks, the seven mammalian isoforms of 14-3-3 regulate many different physiological, as well as pathological processes. 14-3-3 homo- and heterodimers interact with their partner proteins mostly *via* specific phosphorylated binding motifs that bind in the amphipathic binding groove of 14-3-3.^11–13,17,18^ 14-3-3 proteins are highly expressed, especially in the brain where they make up for approximately 1% of the total soluble proteins and play a major regulatory role in brain cellular processes.^12,15,16,19,20^ 14-3-3 has, amongst other, a chaperone-like neuroprotective function to prevent aberrant protein condensation, misfolding, and aggregation. For example, 14-3-3 proteins interact with and modulate several proteins involved in neurodegenerative diseases, such as GFAP in Alexander disease,^21^ α-Synuclein in Parkinson's disease,^16,22^ and Tau in Alzheimer's disease.^2,15,23–30^

In the healthy human brain, soluble (non-pathogenic) phosphorylated Tau (p-Tau) is abundantly present and even enriched during neurodevelopment.^6,31^ This indicates that there is an efficient, protective molecular mechanism that can prevent p-Tau from aggregating. The high abundance of 14-3-3 in the human brain suggests a key role in keeping p-Tau in a soluble state. 14-3-3 has been shown to bind to p-Tau, specifically *via* the pS214/pS324 binding motifs (Fig. 1a and b).^29,30^ 14-3-3 binding to those two phosphorylated sites was recently demonstrated by us to inhibit Tau aggregation and LLPS.^30^ Enhancing this protective Tau/14-3-3 PPI *via* stabilization by small-molecule molecular glues,^18,32^ could provide a long-sought novel pharmacological entry to prevent Tau condensation and aggregation.

**Fig. 1:**
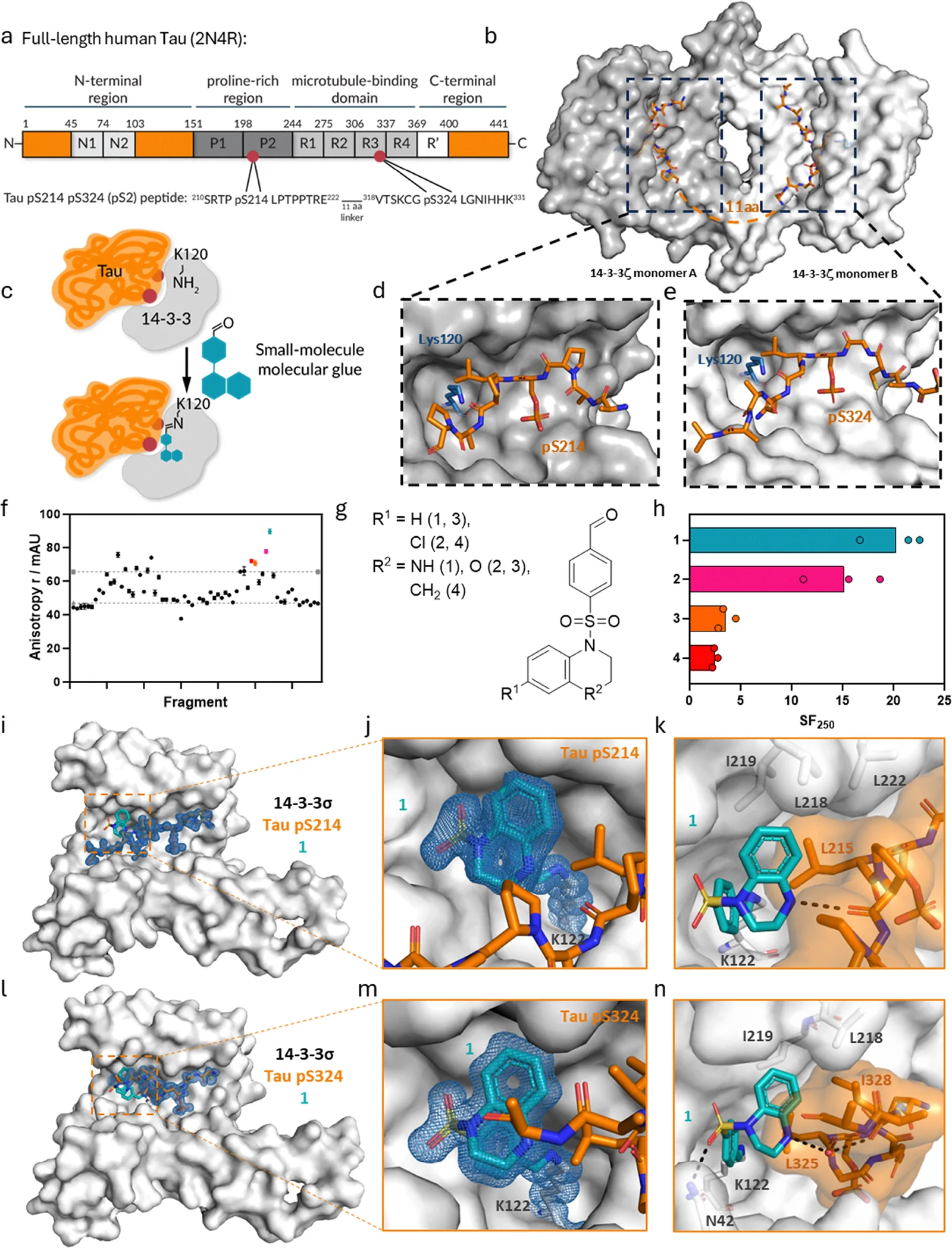
(a) Domain structure of the longest human Tau isoform (2N4R, 441 aa) and sequence of synthetic phospho-peptide Tau-pS214/pS324 pS2 (Tau210-222(pS214)-GGGSGGGSGGG-Tau318-331(pS324). (b) Phosphorylation of Tau residues S214 and S324 enables 14-3-3 binding. Top view of pS214 and pS324 binding sites (orange sticks) into the 14-3-3ζ binding grooves (grey semi-transparent surface) PDB ID: 8QDV. (c) Cartoon representation of a small-molecule molecular glue binding to K120 of 14-3-3ζ (or K122, depending on 14-3-3 isoform), stabilizing the 14-3-3/Tau interaction. (d) and (e) Close-up of the binding grooves of monomer A and B (grey surface) containing the Tau peptide binding sites (orange sticks), including accessibility of K120 (blue sticks). (f) Fluorescent anisotropy (FA) compound screening at 1 mM to fixed concentrations of FITC-labelled Tau (FITC-Tau) pS2 peptide (100 nM) and 14-3-3ζ (2 µM). Deacetylated fusicoccin-A (deAc-FCA, upper grey dashed line) was used as (weak) positive control and DMSO (lower grey dashed line) was used as a negative control. Follow-up hit compounds are color indicated. Data shown as mean ± SD, with *n* = 3 technical replicates. (g) Chemical structures of hit compounds 1-4. (h) Bar plot of stabilization factor (SF_250_, fold-increase in affinity) induced by 250 µM of each compound acting on the 14-3-3ζ/Tau complex. *n* = 3 independent experiments. (i) and (l) Front view of a 14-3-3σ monomer (white surface) bound to Tau pS214 (i) and Tau pS324 (l) phosphopeptides (orange sticks) and compound 1 (blue sticks). 2Fo-Fc electron density maps are contoured at 1σ. PDB IDs: 9GG8 + 9GG7. (j) and (m) Close-up views of 1 in the 14-3-3 binding grooves. 2Fo-Fc electron density maps are contoured at 1σ. (k) and (n) Depiction of polar contacts (black dotted line) and the hydrophobic roof of the 14-3-3 binding groove (white sticks) and water molecules (red spheres).

Reversible-covalent imine tethering has been shown to be a valuable molecular technology to rapidly identify small molecule glues for certain 14-3-3-based PPIs.^33–37^ Such compounds bind reversible-covalently to a specific lysine of 14-3-3, forming an imine bond, located close to the partner protein binding site in the composite PPI binding pocket (Fig. 1c).^36^ (Note that based on the 14-3-3 isoform this lysine is either numbered K120 or K122.) The crystal structure of the binary complex of 14-3-3ζ/Tau pS214-pS324 (pS2 peptide, PDB 8QDV) reveals the accessibility of this K120 of 14-3-3ζ (Fig. 1d and e) in both composite PPI pockets, providing an intriguing starting point for the identification of molecular glues for the 14-3-3/Tau PPI. Herein we show the identification and optimization of such dynamic-covalent aldehyde molecular glues for the 14-3-3/Tau PPI and their potential in modulating reconstituted Tau LLPS.

## Results and discussion

### Identification of molecular glues for the 14-3-3/Tau PPI

A focused library of 67 aldehydes, previously optimised for stabilizing another 14-3-3 PPI,^35^ was screened for stabilization of the 14-3-3ζ/Tau PPI using a fluorescence anisotropy (FA) assay (Fig. 1f). Deacetylated fusicoccin-A (deAc-FCA), a fungal phytotoxin that weakly stabilizes the 14-3-3ζ/Tau PPI 2-fold at 100 µM (SI Fig. S1), was used as positive control. A compound was classified as hit when it had similar or increased anisotropy levels compared to deAc-FCA. Sixteen hits were identified, with the eight most promising compounds being tetrahydroquinoline-based aldehydes. The four most promising tetrahydroquinoline-derived compounds 1–4 (Fig. 1g) were evaluated in a compound-titration assay (SI Fig. S2), with the dose-dependent increases in fluorescence anisotropy demonstrating enhanced 14-3-3ζ/Tau binding induced by the compounds. The time-dependent increase of the anisotropy values reflects the (dynamic) covalent bond formation by the aldehydes.^36^ The compounds were further validated in a FA protein-titration assay, where 14-3-3ζ was titrated to the FITC-Tau pS2 peptide in presence of DMSO (control) or 250 µM compound (SI Fig. S3). The induced left-shifts of the binding curves revealed a 2- to 20-fold PPI stabilization by the compounds (Fig. 1h).

High-resolution X-ray crystal structures (<1.24 Å) were obtained for compound 1 by co-crystallizing the binary 14-3-3σΔC_C38N/Tau pS214 peptide and Tau pS324 peptide complexes (Fig. 1i–n). For both complexes, a continuous electron density was observed for the compound, including the covalent imine bond with K122 of 14-3-3σ (Fig. 1j and m). For the Tau pS214 and pS324 peptides, density was observed for residues T212-T220 and C322-H330, respectively (Fig. 1i and l). For the pS214 peptide, residues T217 and P218 underwent a conformational change compared to the binary crystal structure (PDB 4FL5), enabling accommodation of 1 in the composite pocket (SI Fig. S4). Intriguingly, additional density was observed for Tau residues P219 and T220 in presence of the molecular glue, as compared to the binary complex, reflecting the structural PPI stabilization effects of 1. For the pS324 binding motif, additional density was observed for residues C322 and N327-H330 compared to the binary complex (PDB 5BTV), with the C-terminal residues folding over compound 1. This local fold-back is expected to also be possible in the context of full-length Tau, given the intrinsically disordered nature of the protein, which allows transient, ligand-induced folding of otherwise flexible regions. In both complexes, the annulated benzene ring of 1 is pointing towards the hydrophobic roof (helix 9) of the 14-3-3 binding groove and towards the hydrophobic +1 leucine residue of the pS214 and pS324 peptide (Fig. 1k and n). A polar contact was observed between the amine moiety of the bicycle of 1 and the backbone of P216 of the pS214 peptide (Fig. 1k). Polar contacts were also observed between this amine moiety and the pS324 peptide, for which a small water interaction network was formed inside the 14-3-3 binding groove and the backbone of residue I328 (Fig. 1n). In addition, the sulfonyl-group of 1 formed a polar contact with residue N42 of 14-3-3 (Fig. 1n).

55 new compounds were subsequently designed based on the structural data of compound 1. The composition of the bicyclic ring system and its substituents were varied to explore the stabilization efficiency of the tetrahydroquinoline-derived hits (SI Table S1). This new compound library was prepared using an optimized two-step synthetic route, crucially involving sulfonamide formation followed by deprotection of the aldehyde to provide the final benzaldehyde compounds. The compounds were screened against the 14-3-3ζ/Tau PPI in the FA assay (Fig. 2a) and classified as follow-up hit when demonstrating a greater stabilization than hit compound 1. The resulting five compounds (Fig. 2b) were assessed in a FA compound-titration assay (Fig. 2c and SI Fig. S5). Dose-dependent PPI increases were observed with half-maximum ternary complex formation (CC_50_) values of 44-250 µM (SI Table S1). In the FA protein-titration assay compounds 5–9 induced strong left-shifts of the 14-3-3/Tau binding curve (Fig. 2d). Compounds 5 and 9 showed the strongest stabilizing properties with a SF_250_ (stabilization factor, fold-increase in affinity at 250 µM) of 47 and 53, respectively (Fig. 2e). The enhanced stabilization observed for compounds 5 and 9 may, at least in part, be related to the presence of the bromine substituent, which has previously been associated with improved potency in related compounds. The compounds had a fast onset of PPI stabilization and reached a maximum stabilization of the 14-3-3ζ/Tau complex after 6 h (Fig. 2f), testimony to the two-step stabilization process as observed in previous work.^36^

**Fig. 2:**
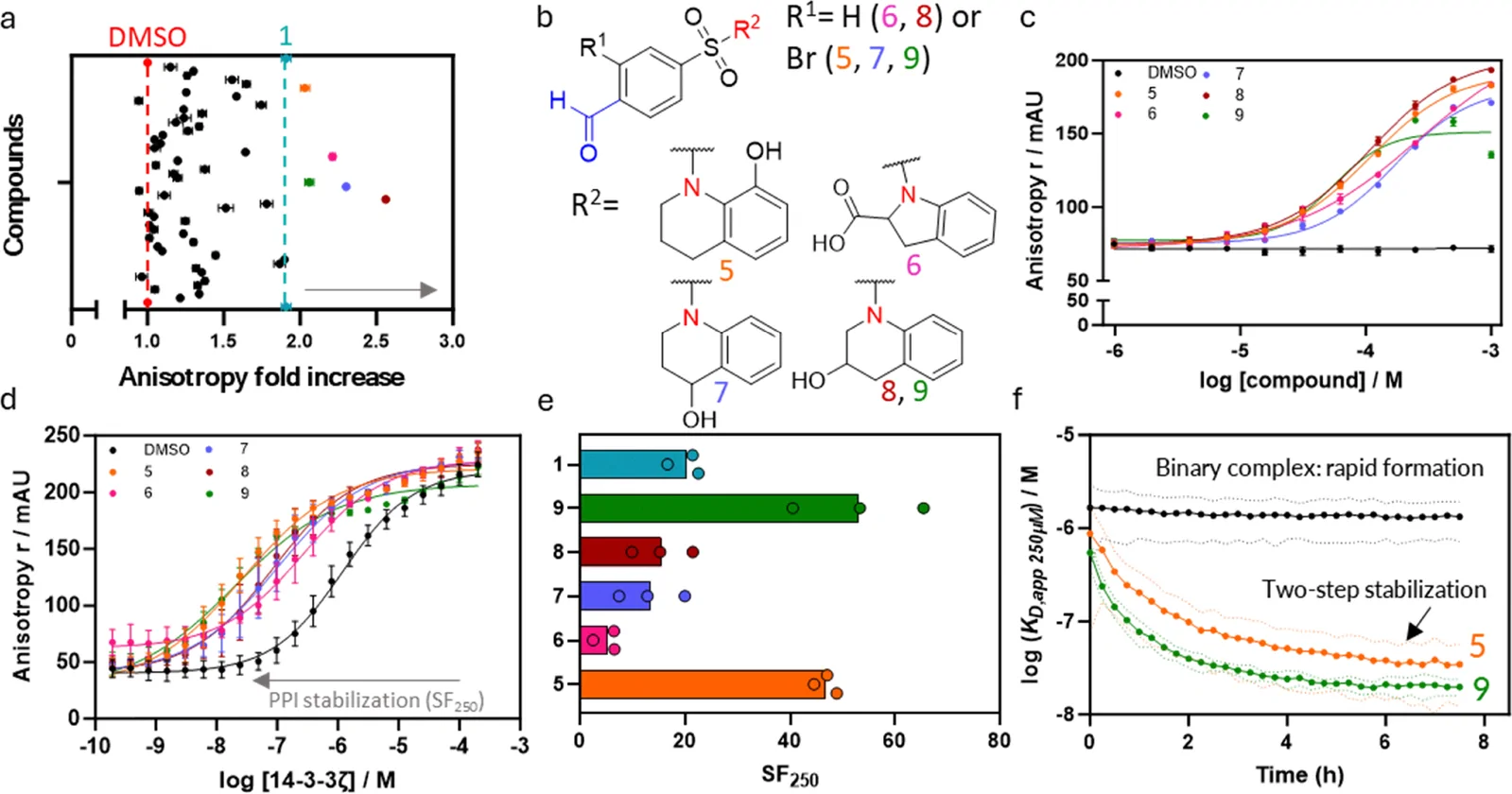
(a) FA compound screening at 500 µM of the new compound library to fixed concentrations of FITC-labelled Tau (FITC-Tau) pS2 peptide (10 nM) and 14-3-3ζ (100 nM) shown as fold-increase in anisotropy relative to DMSO (red). Compound 1 (cyan dashed line) was used as cut-off value for hit compounds (marked in color). (b) Chemical structures of compounds 5–9. (c) Dose–response FA data of 5–9 to 10 nM FITC-labeled pS2 peptide and 100 nM 14-3-3ζ. Data shown as mean ± SD, representative experiment with *n* = 2 technical replicates (see SI Fig. S5 for all replicates). (d) FA assay of 14-3-3ζ titrations to 10 nM FITC-labeled pS2 peptide and 250 µM 5–9. Data shown as mean ± SD, representative experiment with *n* = 3 independent replicates. (e) Bar plot of stabilization factor of the 14-3-3ζ/Tau complex (SF_250_, fold-increase in affinity) induced by 250 µM of compound (*n* = 3 independent experiments). Compound 1 is added for reference and has been measured separately. (f) *K*_D_ of 5 and 9 as function of time. Data shown as mean ± SD, representative experiment with *n* = 3 independent replicates.

### Molecular glue stabilization of full-length 14-3-3/Tau PPI enhances Tau solubility and inhibits Tau LLPS

Molecular glue stabilization of full length (FL) Tau binding to 14-3-3γ was evaluated using a time resolved (TR)-FRET (Fig. 3a) assay. FL-Tau was phosphorylated using Protein Kinase A (PKA-Tau), which also phosphorylates residues S214 and S324,^30^ as confirmed *via* western-blot (SI Fig. S7). 14-3-3γ titration resulted in a binary binding curve with an EC_50_ value of 43 ± 24 nM (Fig. 3b and SI Fig. S8). Upon titration with compounds 5 and 9, an enhanced FRET signal was observed, indicating stabilization of the 14-3-3γ/PKA-Tau PPI by the molecular glues (SI Fig. S9). A significant leftward shift of the 14-3-3γ binding curve was induced by 5 and 9 (Fig. 3c and SI Fig. S10). The hydroxy-variants of 5 and 9 (5-OH and 9-OH) were silent in these titrations, underscoring the dynamic covalent mechanism responsible 14-3-3/PKA-Tau stabilization. Differential scanning fluorimetry (DSF) measurements with both full-length proteins revealed strong PPI melting temperature increases by molecular glues 5 and 9 of 4.8 ± 0.1 °C and 3.4 ± 0.3 °C, respectively, in a clear dose–response manner (Fig. 3d). The biphasic profiles observed at increasing compound concentrations are consistent with the presence of distinct bound states of 14-3-3, corresponding to 14-3-3 alone and bound to Tau in the absence or presence of molecular glue. Again, the 5-OH and 9-OH reference compounds did not affect the PPI (SI Fig. S11).

**Fig. 3:**
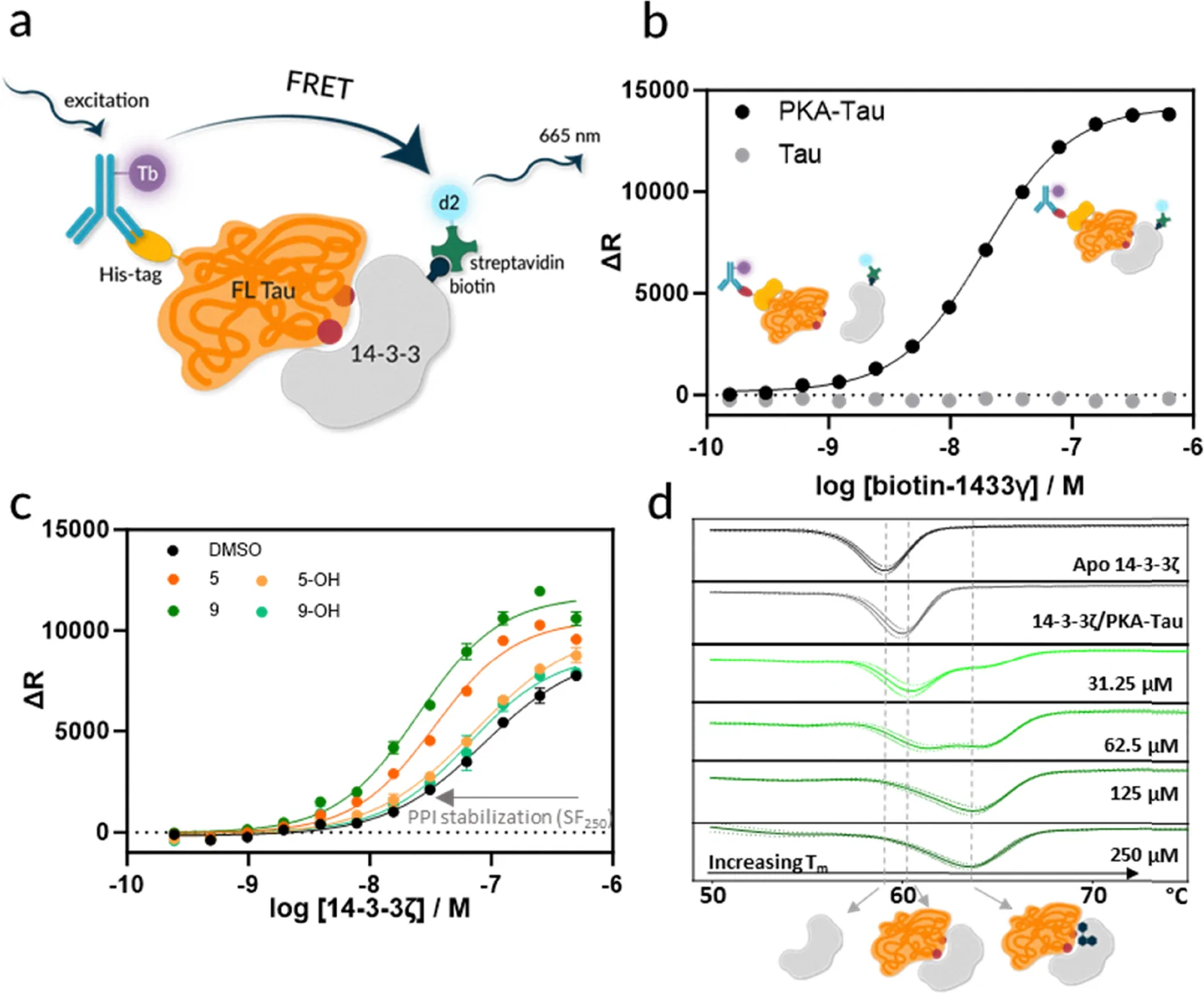
(a) Simplified representation of the 14-3-3/FL Tau TR-FRET assay. Excitation of the Tb donor will only result in FRET to the d2 acceptor fluorophore upon formation of the 14-3-3/FL Tau complex. (b) The ΔRatio (Δ*R*, ratio 665/620 – the background titration) of the dose–response curves of a biotin-14-3-3γ and d2-strep (1 : 4, d2-strep:biotin-14-3-3γ monomer) titration to 40 nM PKA-Tau and non-phosphorylated Tau. The concentration of anti-His Tb was fixed at 0.5 nM. Data represents *n* = 1 (see SI Fig. S8 for one more experiment). (c) Δ*R* of the dose–response curves of biotin-14-3-3γ and d2-strep (1 : 4, d2-strep:biotin-14-3-3γ monomer) titration to 40 nM PKA-Tau in presence of DMSO or 250 µM compound. The concentration of anti-His Tb was fixed at 0.5 nM. Data shown is a representative experiment with *n* = 1 (see SI Fig. S10 for two more experiments). (d) Differential melting curves of 2.5 µM 14-3-3ζ or 2.5 µM 14-3-3ζ + 25 µM PKA-Tau in the presence of increasing concentrations 9. The formation of the three different species can be observed. Data shown as mean ± SD, representative experiment with *n* = 3 independent replicates.

At sub-stoichiometric concentrations 14-3-3 co-condenses with Tau *via* LLPS based on multivalent electrostatic interactions. Phosphorylation of Tau at S214 and S324 leads to stochiometric 14-3-3/Tau binding, which inhibits the assembly of Tau into biomolecular condensates.^30^ Compounds that enhance the 14-3-3/Tau PPI could thus potentially reduce Tau condensation. As such, compound 9, identified as the most promising molecular glue from our campaign, was evaluated regarding its potential to modulate PKA-Tau condensation (Fig. 4a). Fluorescence microscopy visualization of 14-3-3/PKA-Tau co-condensates revealed that an increasing concentration of molecular glue 9, at constant Tau and 14-3-3 concentrations, resulted in a decrease in LLPS (Fig. 4b). Quantification of condensate surface coverage (Fig. 4f and SI Fig. S12), revealed the dose-dependence of 9. Importantly, reference compound 9-OH did not elicit an effect on the LLPS, confirming the molecular glue mechanism (Fig. 4c and g). The condensation of Tau alone was not affected by 9, demonstrating its 14-3-3 PPI-mediated mechanism on LLPS inhibition (Fig. 4d and h). The crucial role of Tau phosphorylation at pS214 and pS324 in interplay with 9 was confirmed using a Tau variant in which S214 and S324 were replaced by alanine residues (TauS2A) (SI Fig. S7); PKA-phosphorylated TauS2A formed condensates which were not suppressed with molecular glue 9 (Fig. 4e and i). A small increase in surface coverage was even observed at the high compound concentration (Fig. 4i).

**Fig. 4:**
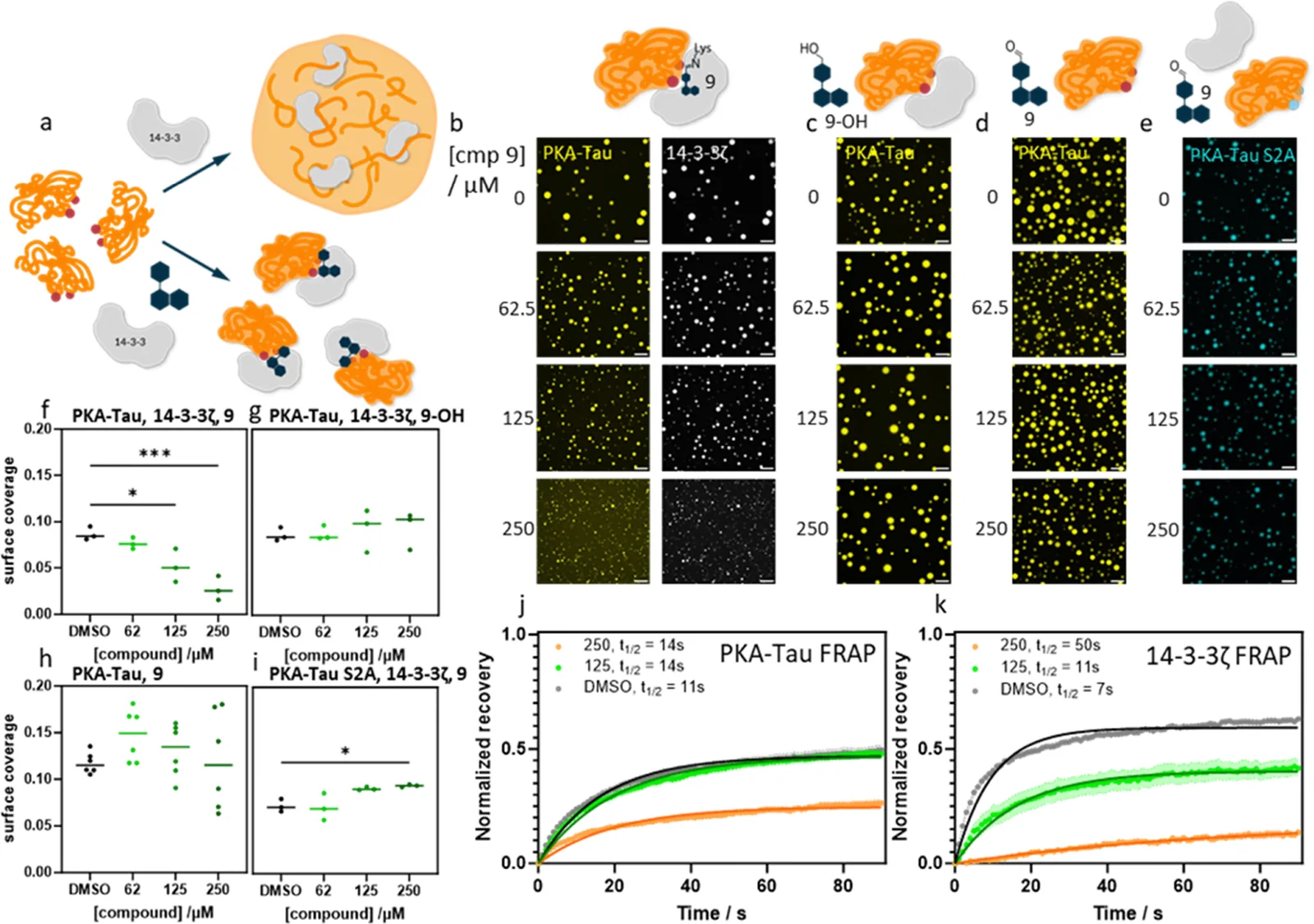
(a) Schematic representation of the effect of molecular glue mediated 14-3-3/Tau stabilization on LLPS. Tau normally co-condenses with 14-3-3, but when phosphorylated and upon addition of the molecular glue, the stabilized 14-3-3/Tau interaction will keep Tau in its soluble state, preventing LLPS. (b)–(e) Representative fluorescence microscopy images of 10 µM PKA-Tau (b), (c), and (d) or PKA-TauS2A (e) +7.5 µM 14-3-3ζ (b), (c) and (e) with increasing 9 (b, d, e) or 9-OH (c) concentrations (0, 62.5, 125, 250 µM) in 25 mM HEPES, 1 mM DTT, pH 7.4 buffer in the presence of 5% (w/v) PEG. 1% Tau labeled with DyLight561, 1% TauS2A labeled with DyLight488, 1% 14-3-3ζ labeled with DyLight405. Scale bars = 10 µm. (f)–(i) Quantification of condensate surface coverage. Data shown as mean ± SD. One-way ANOVA test. Data points represent the average surface coverage of independent replicates derived from 3 individual images per data point and condition. (j) and (k) FRAP of 10 µM PKA-Tau and 7.5 µM 14-3-3ζ with increasing concentrations of 9. Tau labeled with 1% DyLight561 and 14-3-3ζ labeled with 1% DyLight405. Data presented as mean ± SEM; *n* = 15–30 condensates per condition. Solid lines show one phase association fit to the data (dots).

Fluorescence recovery after photobleaching (FRAP) measurements were performed on the 14-3-3/PKA-Tau condensates. FRAP was measured for both 14-3-3 and Tau in condensates, a proxy for their molecular mobility (Fig. 4j, k and SI Fig. S13). The mobile fraction of both proteins inside condensates was reduced upon treatment with the molecular glue. This indicates that the presence of compound 9 led to an immobilization, or “locking”, of both proteins in the protein network inside condensates. This effect was strongest for 14-3-3, for which also a significant increase in half-time (*t*_1/2_) was observed, probably due to the excess of PKA-Tau protein (more PKA-Tau than needed for stoichiometric binding to 14-3-3). It should be noted that these experiments only observed 14-3-3 and PKA-Tau molecules present in the liquid-condensed phase and not the stochiometric complexes that had already dissolved out of condensates into the surrounding light phase.

## Conclusions

Tau condensation *via* LLPS is a precursor to the formation of pathological Tau species, and the concentration- and phosphorylation-state-dependence of this process *in vivo* remains incompletely understood. Compounds that modulate and inhibit Tau condensation therefore represent valuable chemical tools to interrogate this relationship and, ultimately, have potential as therapeutic approaches to mitigate Tau aggregation and toxicity in neurodegenerative diseases.

Here, reversible covalent molecular glues were identified that stabilize the 14-3-3/Tau PPI. Site-specific binding of these compounds to the composite PPI interface was validated using X-ray co-crystallography, and their stabilizing effects were confirmed through a set of biochemical assays. TR-FRET and DSF assays confirmed the effective PPI stabilization effects of these molecular glues also on full-length Tau. Biomolecular condensate studies highlighted that molecular glue compound 9 enhanced Tau solubility and inhibited Tau LLPS. The FRAP studies further testify to the strengthening of the 14-3-3/Tau PPI by the molecular glues also within the condensate and suggest that 14-3-3/Tau stabilization results in a complex interplay of both soluble and condensate-sequestered 14-3-3/Tau/molecular glue species.

The molecular glues reported here modulate Tau LLPS *via* a druggable molecular mechanism at the composite 14-3-3/Tau interface and provide starting points for chemical probes to dissect the concentration- and phosphorylation-dependent aspects of Tau aggregation. The current compounds are still fragment-like in size, but their site-specific binding mode establishes a tractable basis for further activity and selectivity optimization. More broadly, this work illustrates a drug development strategy for modulating, 14-3-3 regulated, protein condensation, offering conceptual opportunities for drug development in tauopathies and, potentially, other protein misfolding diseases.

## Author contributions

MCMvdO, LR and FMZ were in the lead with experimental planning and execution. MCMvdO, LR, FMZ, AO, TRvdW, FMAvB, MAMP, CJAV, and TWvV performed experiments. PJC provided guidance and scientific input, CO, SW, and LB provided guidance, scientific input and funding resources, MCMvdO and LB wrote the manuscript with input from all authors.

## Conflicts of interest

CO and LB are co-founders of Ambagon Therapeutics. The other authors declare no conflicts.

## Supplementary Material

CB-007-D6CB00156D-s001

## Data Availability

The data that support the findings of this study are available in the supplementary information (SI) of this article. Specifically, the SI contains experimental procedures, supporting Tables S1–S3, supporting Fig. S1–S22, as well as all the structures and characterization of the molecules described in this manuscript. See DOI: https://doi.org/10.1039/d6cb00156d. Crystallographic data presented in this manuscript has been deposited on the open access repository RSCB Protein Data Bank (https://www.rcsb.org/) *via* the PDB codes 9GG7, 9GG8, and 9GGA.
