# Supplementary material for: 14-3-3/Tau molecular glues modulate in vitro tau condensation
Source: RSC Chem Biol. 2026 Aug 28;7(10):2091–7. doi: 10.1039/d6cb00156d (PMC13551062; doi:10.1039/d6cb00156d)
Supplement: Supplementary file 1 [file CB-007-D6CB00156D-s001.pdf]

Supplementary Information (SI) for RSC Chemical Biology.
This journal is © The Royal Society of Chemistry 2026

14-3-3/Tau Molecular Glues Modulate in vitro Tau Condensation

Supplementary Information

Experimental section

Solvents

All solvents used in this work were purchased from Biosolve and were used without further purification. Water was purified by a Milipore purification train. Deuterated solvents were obtained from Cambridge Isotope Laboratories.

Reagents

Reagents were commercially available and were supplied by Sigma-Aldrich, TCI, BLDpharm, Fluorochem, and abcr GmbH.

Thin Layer Chromatography

Reaction progress was monitored by thin-layer liquid chromatography using Merck precoated silica gel plates (60F-254). Compounds were visualized using ultraviolet light irradiation at 254 nm.

Silica Gel Flash Chromatography

Flash column chromatography was performed on a *Biotage Isolera One* using *Büchi FlashPure EcoFlex Silica Cartridges* with 40-63 μm particles. For flash column chromatography, dry loading or liquid loading was performed.

NMR Spectroscopy

^{1}H- and ^{13}C{^{1}H}-NMR spectra were recorded using a *Bruker Avance* 400 MHz spectrometer. Proton spectra are referenced to the internal standard tetramethyl silane (TMS). Carbon spectra are referenced to tetramethyl silane or the solvent peak. NMR spectra are reported as follows: chemical shift, multiplicity

(s = singlet, d = doublet, t = triplet, q = quartet, m = multiplet, dd = doublet of doublet, td = triplet of doublets), coupling constant (*J*) in Hertz (Hz) (if applicable) and integration (proton spectra only). Residual peaks of the particular solvent were used as an internal standard (δ_H($CHCl_3$) = 7.26 ppm, δ_C($CDCl_3$) = 77.16 ppm; δ_H(DMSO-d_5) = 2.50 ppm, δ_C(DMSO-d_6) = 39.52 ppm).

14-3-3ζ and 14-3-3σΔc protein expression and purification (FA, DSF, LLPS, FRAP & Crystallography)

A pPROEX HTb expression vectors encoding the human 14-3-3 zeta (14-3-3ζ) and the human 14-3-3 protein sigma truncated after T231 (14-3-3σΔc, to reduce flexiblity) with an N-terminal his6-tag were transformed by heat shock into NiCo21 (DE3) competent cells. A single colony was selected and cultured overnight in 35 mL LB medium (100 μg/mL ampicillin) at 37°C. After overnight incubation, cultures were transferred to 2 L TB medium (100 μg/mL ampicillin, 1 mM MgCl2) and incubated at 37°C, 140 rpm until an OD600 of 0.6-1.2 was reached. Protein expression was then induced with 0.4 mM isopropyl-β-d-thiogalactoside (IPTG), and cultures were incubated overnight at 18°C, 140 rpm. Cells were harvested by centrifugation (8600 rpm, 20 minutes, 4 °C) and resuspended in lysis buffer (50 mM Hepes, pH 8.0, 300 mM NaCl, 12.5 mM imidazole, 5 mM MgCl2, 2 mM βME) containing cOmplete™ EDTA-free protease inhibitor cocktail tablets (1 tablet/ 100 ml lysate) and benzonase (5 μl/ 100 ml). After lysis using a C3 EmulsiflexC3 homogenizer (Avestin), the cell lysate was cleared by centrifugation (20000 rpm, 30 minutes, 4 °C) and purified using Ni2+-affinity chromatography (HisTrap high performance cartridges, Cytiva). Typically two 5 mL columns were used for a 2 L culture in which the lysate was loaded on the column, washed with 10 CV wash buffer (50 mM Hepes, pH 8.0, 300 mM NaCl, 25 mM imidazole, 2 mM βME), and eluted with several fractions (2-4 CV) of elution buffer (50 mM Hepes, pH 8.0, 300 mM NaCl, 250 mM imidazole, 2 mM βME). Fractions containing the 14-3-3 protein were combined and dialyzed into 25 mM HEPES pH 8.0, 200 mM NaCl, 10 mM MgCl2, 2 mM βME, followed by dialysis into 25 mM HEPES, 100 mM NaCl, 10 mM MgCl2, 500 μM TCEP (adjusted to pH 8.0). Dialyzed protein was concentrated to ~50 mg/mL, analyzed by Q-Tof MS, and aliquots were flash-frozen for storage at −80 °C. For 14-3-3σΔc, 1 mg TEV per 100 mg purified protein was added to the dialysis to remove the purification tag. The cleaved sample was then again loaded on a 10 mL Ni-NTA column to separate the cleaved product from the expression tag and residual uncleaved protein. The flowthrough was loaded on a Superdex 75 pg 16/60 size exclusion column (GE Life Sciences) using 25 mM HEPES, 100 mM NaCl, 10 mM MgCl2, 500 μM TCEP (adjusted to pH = 8.0) as running buffer. Fractions containing the 14-3-3 protein were pooled and concentrated to ~60 mg/mL, analyzed Q-Tof MS, and aliquots were flash-frozen for storage at −80°C.

Protein expression and purification of biotin-14-3-3 constructs. (TR-FRET)
A pETDuet-1 expression vector encoding the human 14-3-3 protein with an N-terminal His-AVI-tag was transformed in NICO21 (DE3) competent cells by heat shock. The transformed bacteria were precultured in 8 mL LB medium (100 µg/mL ampicillin) and cultured overnight at 37°C, 250 rpm. The preculture was transferred to 1L TB medium (100 µg/mL ampicillin, 1 mM MgCl2) and incubated at 37°C, 140 rpm until an OD600 of 0.6-1.2 was reached. 0.5 mM biotin in PBS was added to allow biotinylating.

Subsequently, protein expression was induced by the addition of 0.4 mM IPTG and the cultures were incubated overnight at 37°C, 140 rpm. Cells were harvested by centrifugation (10.000 xg, 10 minutes, 4°C) and were resuspended in lysis buffer (50 mM Hepes pH=8.0, 300 mM NaCl, 12.5 mM imidazole, 5 mM MgCl2, 2 mM BME, 10 mL lysis buffer per gram of pellet), containing cOmpleteTM EDTA-free Protease Inhibitor Cocktail tables (1 tables/100 mL) and benzonase (5 µL/100 mL). Lysate was obtained using the Emulsiflex-C3 homogenizer (Avestin) and the protein-containing supernatant was obtained by centrifugation at 20.000 rpm, 30 minutes, 4°C. The supernatant was loaded onto a Ni-NTA affinity column (HisTrap high performance cartridges, Cytiva), pre-equilibrated with wash buffer (50 mM Hepes pH=8.0, 300 mM NaCl, 40 mM imidazole, 2 mM BME). After washing the column with wash buffer, protein bound to the column was eluted with 200 mM imidazole. The eluted protein was added to a dialysis bag (10.000 Da pores) and placed in 1L dialysis buffer (25 mM Hepes pH=8.0, 200 mM NaCl, 2 mM BME). After overnight dialysis, the dialysis bag was transferred into the final buffer (25 mM Hepes pH=8.0, 100 mM NaCl, 0.5 mM TCEP) and dialyzed again overnight.

Protein expression and purification of FL (His-SUMO-)Tau constructs. (TR-FRET, DSF)
BL21 competent Escherichia coli cells (DE3) were transformed with the pET28a(+) vector containing the HS-Tau sequence. The transformed BL21 E. coli cells were precultured in 8 mL LB medium containing 1000x kanamycin and were grown overnight at 37°C, 250 rpm. The preculture was transferred to 1L TB medium (50 µg/mL kanamycin) and incubated at 37°C, 140 rpm until an OD600 of 0.6-1.2 was reached. Subsequently, protein expression was induced by the addition of isopropyl β-D-1-thiogalactopyranoside (IPTG) to a final concentration of 0.1 mM, followed by overnight incubation at 18 ˚C, 140 rpm. Cells were harvested by centrifugation (10.000 xg, 10 minutes, 4°C) and were resuspended in lysis buffer (50 mM Hepes pH=8.0, 300 mM NaCl, 20 mM imidazole, 2 mM β-mercaptoethanol (BME), 1 tablet of protease inhibitors and 5-10 µL benzonase, 10 mL lysis buffer per gram of pellet). Lysate was obtained by sonication (protocol of 5 minutes, 5 seconds pulse on, 5 seconds pulse off, amplitude of 70%) using the Qsonica Q500 and the protein-containing supernatant was obtained by centrifugation at 20.000 rpm, 30 minutes, 4°C. The supernatant was loaded onto a nickel-nitrilotriacetic acid (Ni-NTA) affinity column (HisTrap high performance cartridges, Cytiva), pre-equilibrated with wash buffer (50 mM Hepes pH=8.0, 300 mM NaCl, 40 mM imidazole, 2 mM BME). After washing the column with wash buffer, protein bound to the column was eluted with 200 mM imidazole. The eluted protein was added to a dialysis bag (10.000 Da pores) and placed in 1L dialysis buffer (25 mM Hepes Ph=8.0, 200 mM NaCl, 2 mM BME). After overnight dialysis, the dialysis bag was transferred into the final buffer (25 mM Hepes pH=8.0, 100 mM NaCl, 0.5 mM tris(2-carboxyethyl)phosphine (TCEP)) and dialyzed again overnight. For the His-SUMO cleaved constructs, the eluted protein was added to a dialysis bag (10.000 Da pores), placed in 1L dialysis buffer (25 mM Hepes pH=8.0, 200 mM NaCl, 2 mM BME), and SUMO hydrolase was added in a 1:100 ratio (SUMO hydrolase:FL Tau protein). After overnight dialysis, the mixture was loaded onto a Ni-NTA affinity column (HisTrap high performance cartridges, Cytiva), pre-equilibrated with wash buffer (50 mM Hepes pH=8.0, 300 mM NaCl, 40 mM imidazole, 2 mM BME) and FL Tau was purified from the cleaved His-SUMO tag and the His-tag containing SUMO hydrolase. Purified protein was again added to a dialysis bag (10.000 Da pores) and placed in 1L dialysis buffer (25 mM Hepes pH=8.0, 200 mM NaCl, 2 mM BME). After overnight dialysis, the dialysis bag was transferred into the final buffer (25 mM Hepes pH=8.0, 100 mM NaCl, 0.5 mM TCEP) and dialyzed again overnight.

In vitro phosphorylation of Tau
(HS-)Tau was incubated with His-PKA-Strep (ratio 1:200 (Tau:His-PKA-strep)) at 30°C, overnight, in phosphorylation buffer (25 mM HEPES pH 8.0, 100 mM NaCl, 0.5 mM TCEP, 5 mM MgCl2) containing ATP (10x excess over HS-Tau). The sample was loaded onto a Strep-Tactin®XT 4Flow affinity column, pre-equilibrated with wash buffer (100 mM Tris pH 8.0, 150 mM NaCl, 1 mM EDTA) to purify HS-pTau from His-PKA-Strep. Purified HS-pTau was captured during the washing step, and His-PKA-Strep was eluted from the column with 50 mM biotin. Purified protein was added to a dialysis bag (10.000 Da pores) and placed in 1L final buffer (25 mM Hepes pH=8.0, 100 mM NaCl, 0.5 mM TCEP) to dialyze overnight.

Protein expression, purification and phosphorylation of FL Tau constructs. (LLPS, FRAP)
Recombinant human full-length Tau (2N4R, hTau40) was expressed in E. coli BL21 Star (DE3) (Invitrogen) cells as previously described[38] and purified and phosphorylated following an established protocol[39,40]. All recombinant purified and further modified proteins were stored aliquoted at −80°C.

Tau peptides
Acetylated and FITC-labeled Tau peptides for FA, crystallography and TR-FRET were purchased from GenScript Biotech Corp. Sequences were:
Tau pS2 peptides:
RTP{pSER}LPTGGGSGGGSGGGSKCG{pSER}LGNIHHK (used in the initial screen and FCA titrations)
SRTP{pSER}LPTPPTREGGGSGGGSGGGVTSKCG{pSER}LGNIHHK (used in all other experiments)
Tau pS214: SRTP{pSER}LPTPPTRE
Tau pS324: VTSKCG{pSER}LGNIHHK

Western Blot
Tau variants (0.5 µg protein) and neuronal lysates (10 µg protein), was separated by SDS-PAGE (NuPage 4-12% Bis-Tris, Invitrogen) and blotted onto a nitrocellulose membrane. The membrane was blocked with 3% BSA in PBS containing 0.05% Tween (PBS-T) at room temperature for 1 h, followed by the incubation with primary anti-phospho-Tau antibodies (rabbit anti-pS214 Tau, Abcam, ab170892 (1:1000); rabbit anti-pS324 Tau, Abcam, ab109401 (1:5000)) or anti-total Tau antibody (rabbit anti-human Tau, Dako, #A0024 (1:5,000)) diluted in 3% BSA in PBS-T overnight at 4°C shaking. After washing with PBS-T, the membrane was incubated with HRP-conjugated secondary antibody (goat anti-rabbit HRP, BioLabs 7074P2) in 3% BSA in PBS-T shaking for 1-2 h at room temperature, washed in PBS-T, and then developed using a chemiluminescent substrate (LumiGLO, Peroxide, 7003, Cell Signaling) and an imaging device (Fusion Fx7).

Fluorescence Polarization – compound screening
Compound screening assays were performed by mixing compound (1 mM, 1% DMSO)) to fixed concentrations of FITC-labelled Tau (FITC-Tau) pS2 peptide (100 nM) and 14-3-3ζ (2 µM) in FA buffer (10 mM HEPES pH 7.4, 150 mM NaCl, 50 µM TCEP, 0.1% (v/v) Tween20, 0.1% (w/v) BSA). Assay mixes were made in polystyrene low-volume 384-well plates (Corning #4514, Black Round Bottom). Measurements were performed after overnight incubation, using a Tecan SPARK plate reader at room temperature (λex: 485±20 nm; λem: 535±25 nm; mirror: automatic; flashes: 30; settle time: 1 ms; gain: optimal; Z-position: calculated from well). Wells containing only fluorescein labelled peptide were used to set as G-factor calibrated from these wells. All data were analyzed using GraphPad Prism (10.0.1) and comounds were classified as hit when having similar or increased anisotropy levels compared to deAc-FCA (positive control). Data shown is the mean and standard deviation of technical triplicates.

Fluorescence Polarization – compound titration
Compound titration assays were performed by titrating compounds (in 1% DMSO, starting at 1000 µM) in a 2-fold dilution series to preformed complex of fluorescein-labeled Tau pS2 (10 nM) and 14-3-3ζ (100 nM) (which represents approximately 20% bound Tau pS2 peptide to 14-3-3ζ to allow further complex formation by addition of fragments) in FA buffer (10 mM HEPES pH 7.4, 150 mM NaCl, 50 µM TCEP, 0.1% (v/v) Tween20, 0.1% (w/v) BSA). Dilution series were made in polystyrene low-volume 384-well plates (Corning #4514, Black Round Bottom). Measurements were performed after overnight incubation, using a Tecan SPARK plate reader at room temperature (λex: 485±20 nm; λem: 535±25 nm; mirror: automatic; flashes: 30; settle time: 1 ms; gain: optimal; Z-position: calculated from well). Wells containing only fluorescein labelled peptide were used to set as G-factor calibrated from these wells. All data were analyzed using GraphPad Prism (10.0.1) and fitted with a four-parameter logistic model (4PL) to determine binding affinities (dissociation constant, K_D). All data shown is the mean and standard deviation of technical duplicate is plotted, assay performed in a scientific duplicate for hit compounds.

Fluorescence Polarization – 14-3-3 titrations in the presence of compound
To characterize the affinity of the Tau pS2 peptide for 14-3-3 in the presence of compound, 14-3-3ζ was titrated in a 2-fold dilution series starting at 400 µM. 14-3-3 was added to 10 nM of fluorescein-labeled peptide (Tau pS2) in the presence of 250 µM compound (1% DMSO). The assays were performed in a similar way as described above. Data shown is the mean and standard deviation of a scientific triplicate.

Differential Scanning Fluorimetry (DSF)
DSF was performed using 40 µl samples containing 2.5 µM 14-3-3ζ, 25 µM PKA-Tau, 0-250 µM compound (1% DMSO) and 5x ProteoOrange (Lumiprobe, 5000x stock in DMSO) in 10 mM HEPES, 150 mM NaCl, 50 µM TCEP (pH 7.4). Measurements were performed after overnight incubation. The samples were heated from 35°C to 79°C at a rate of 0.3°C per 15 s in a CFX96 Touch Real-Time PCR Detection System (Bio-Rad). Fluorescence intensity was determined using excitation (λem=525/20 nm) and emission (λex=570/20 nm) filters. Based on these melting curves, the negative derivative melting curve is obtained from which the melting temperature Tm was determined. All described melting temperatures are based on three independent experiments, from which the average and standard deviations were determined.

X-ray crystallography data collection and refinement
14-3-3σΔC(-C38N, Cys38 mutated to an Asn) and the acetylated TaupS214 and pS324 peptides were dissolved in complexation buffer (20 mM Sodium HEPES pH 7.5, 2 mM $MgCl_2$, and 1.5 mM TCEP) and mixed in a 1:2 molar stoichiometry (protein:peptide) at a final protein concentration of 12.5 mg/ml. The complex was set up for sitting-drop crystallization and crystals were grown within one week at 4°C in 0.19 M Calcium chloride dihydrate, 0.095 M Sodium HEPES pH 7.1, 5% glycerol, and 28% PEG 400. Crystals were fished and flash-cooled in liquid nitrogen. X-ray diffraction (XRD) data was collected at the European Synchrotron Radiation Facility (ESRF) beamline MASSIF-1 (ID30A-1) and ID30B, Grenoble, France.

DIALS[41] (version 3.18.0) was used to index and integrate the diffraction data. The data was further processed using the CCP4i2 suite (version 8.0.017)[42]. Scaling was performed using AIMLESS.[43,44] PHASER[45] was used for phasing, using a binary 14-3-3σΔC-C38N/MAPT complex as a template. REFMAC[46,47] (version 5.7.0009) was used for initial structure refinement. Correct peptide sequences were modelled in the electron density in COOT (version 0.9.8.93).[48] A three dimensional structure of each compound was generated using AceDRG7[49], which was thereafter built in based on visual inspection Fo-Fc and 2Fo-Fc electron density map. Alternating cycles of model building and refinement were performed using COOT and BUSTER[50]. Figures were generated with PyMOL (version 2.5.2). 2Fo-Fc electron density maps were contoured at 1σ. See SI Table S2 and for XRD data collection, structure determination, and refinement statistics. The structures were submitted to the PDB with IDs: 9GG7, 9GG8 and 9GGA.

Homogeneous Time Resolved Fluorescence – 14-3-3 titrations
Biotin-14-3-3γ and d2-strep (CisBio Bioassays, 610SADLA)(ratio 1:4, d2-strep:biotin-14-3-3 monomer) were titrated in a 2-fold series to 40 nM HS-(p)Tau and 0.5 nM anti-His Tb (CisBio Bioassays, 61HISTLA) in TR-FRET buffer (10 mM Hepes, pH 7.5, 150 mM NaCl, 100 µM chaps, 5 mM DTT, 0.1% (w/v) BSA). Dilution series were made in Corning white low volume, low binding, 384-well plates at final volumes of 5 or 10 µL. Measurements were performed 1 hour after plate preparation, using the Tecan FP Spark plate reader at room temperature (ex. = 340 nm, em. 620 nm and 665 nm, time delay: 150 µs, gain: optimal, Z-position: calculated from well). Data represents n=1, SI Figure S8 for another replicate.

Homogeneous Time Resolved Fluorescence – compound titrations.
Compound was titrated in a 2-fold series to 150 nM biotin-14-3-3γ/σ and d2-strep (CisBio Bioassays, 610SADLA)(ratio 1:4, d2-strep:biotin-14-3-3 monomer), 40 nM HS-(p)Tau and 0.5 nM anti-His Tb (CisBio Bioassays, 61HISTLA) in TR-FRET buffer (10 mM Hepes, pH 7.5, 150 mM NaCl, 100 µM chaps, 5 mM DTT, 0.1% (w/v) BSA, DMSO to a final percentage of 2%). A titration with DMSO was performed as a reference. Dilution series were made in Corning white low volume, low binding, 384-well plates at final volumes of 5 µL. Measurement was performed after overnight incubation of the prepared plate, using the Tecan FP Spark plate reader at room temperature (ex. = 340 nm, em. 620 nm and 665 nm, time delay: 150 µs, gain: optimal, Z-position: calculated from well). Data shown for three individually performed experiments.

Homogeneous Time Resolved Fluorescence – 14-3-3 titrations in presence of compound.
Biotin-14-3-3γ/σ and d2-strep (CisBio Bioassays, 610SADLA)(ratio 1:4, d2-strep:biotin-14-3-3 monomer) were titrated in a 2-fold series to 40 nM HS-(p)Tau and 0.5 nM anti-His Tb (CisBio Bioassays, 61HISTLA) in presence of DMSO or 250 µM compound in TR-FRET buffer (10 mM Hepes, pH 7.5, 150 mM NaCl, 100 µM Chaps, 5 mM DTT, 0.1% (w/v) BSA, DMSO to a final percentage of 1%). Dilution series were made in Corning white low volume, low binding, 384-well plates at final volumes of 5 µL. Overtime measurements were performed at every hour for 15 hours after plate preparation, using the Tecan FP Spark plate reader at room temperature (ex. = 340 nm, em. 620 nm and 665 nm, time delay: 150 µs, gain: optimal, Z-position: calculated from well). Data represents n=1, SI Figure S10 for another replicate.

Homogeneous Time Resolved Fluorescence – Data analysis
All TR-FRET data was analyzed using GraphPad Prism (10.1) for Windows and fitted using a four-parameter logistic model:

$$y = A_1 + \frac{A_2 - A_1}{1 + \frac{EC_{50}^{p}}{x^{p}}}$$

y is the Ratio 665/620 or ΔR signal, A1 is the bottom asymptote, A2 is the top asymptote, p is the hill slope, x is the ligand concentration, and the EC_{50} is the half-maximal complex concentration.

Fluorescent labeling of Tau and 14-3-3ζ
Fluorescent labelling of Tau and 14-3-3ζ protein for microscopy assays was done using amine-reactive DyLight405 (14-3-3), DyLight488 (PKA-TauS2A) or DyLight561 (PKA-Tau)-NHS ester (Thermo Scientific) following the manufacturer instructions and the established protocol [39].

Condensate formation and fluorescence microscopy
Prior to condensation assays, all proteins used were dialyzed against 25 mM HEPES, 10 mM NaCl, 1 mM DTT, pH7.4 and stored at -80°C. LLPS of PKA-Tau (10 µM, 1% DyLight561-Tau) or PKA-S2ATau (10 µM, 1% DyLight488-Tau) with 14-3-3ζ (7.5 µM, 1% DyLight405-14-3-3ζ) variants was performed in 25 mM HEPES, 1 mM DTT, pH7.4 buffer (final NaCl concentration in sample 1-5 mM). The Tau/14-3-3 complexes were overnight incubated with compound (0-250µM) after which LLPS was induced by the addition of 5 % (w/vol) PEG-8000. Directly after LLPS induction, 10 µl samples were pipetted onto a optically clear flat-bottom, ultra-low-attachment-coated 384-well microplate for imaging (Revvity). Imaging plates were immediately closed to avoid evaporation of the LLPS sample. Imaging was performed 1 h after droplet formation on a spinning disk confocal microscope (CSU-X, Nikon) equipped with 20x air or 60x oil objective.

Fluorescence recovery after photobleaching (FRAP)
Condensates (containing 1%Tau-DyLight561 and 1%14-3-3ζ-DyLight405) were imaged at 1 sec intervals for 90 sec (round ROIs, cross sections 1-2 µm) with a 405 nm laser or a 561 nm laser (100% intensity, 2-5 sec until the signal is reduced by at least 70%). In each field of view, same-sized background ROI (outside condensates) and non-bleached reference-ROI (inside different condensates) were measured. All FRAP measurements were background corrected and normalized to the background corrected reference signal. FRAP experiments were performed on a spinning disk confocal microscope (CSU-X, Nikon) using a 60x oil objective.

General synthetic procedure
A solution of amine (1 eq.) and **11** (1.2 eq.), and EtN*i*Pr_2 (2.4 eq.) in CH_2Cl_2 (0.1 mol/L) was stirred 16 h at room temperature. Aqueous HCl (1 M, 10 mL) was added and the aqueous layer was extracted with EtOAc (4 x 20 mL). The combined organic extracts were treated with Na_2SO_4 and evaporated. The residue was dissolved in MeOH (0.5 mol/L). K_2CO_3 (1.5 eq.) was added and the mixture was stirred 1 h at room temperature. The volatiles were removed under reduced pressure. Aqueous NaCl (sat., 10 mL) was added and the aqueous layer was extracted with EtOAc (4 x 20 mL). The combined organic extracts were treated with Na_2SO_4 and evaporated. Flash column chromatography (silica, heptane/EtOAc) afforded the desired benzaldehyde.

General buildingblocks

3-bromo-4-methylbenzenesulfonyl chloride

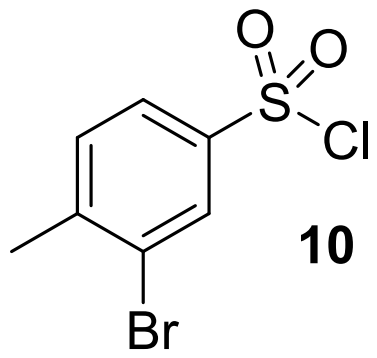

10

To a solution of 4-methylbenzenesulfonyl chloride (67.2 g, 353 mmole, 1 eq.) in 600 mL 95% sulfuric acid was slowly added *N*-bromosuccinimide (75.3 g, 423 mmole, 1.2 eq). The solution was stirred for 16 h at room temperature. The reaction was quenched by the addition of a saturated $NaHCO_3$ solution (10 L, 0°C). The precipitate was collected by filtration, washed with water (3 times 200 mL) and dried in vacuo to yield 3-bromo-4-methylbenzenesulfonyl chloride as a white solid (94.4 g, 350 mmole, 99% yield). **^{1}H NMR** (399 MHz, $CDCl_3$) δ 8.19 (s, 1H), 7.87 (dd, *J* = 8.2, 2.1 Hz, 1H), 7.48 (d, *J* = 8.2 Hz, 1H), 2.53 (s, 3H). **^{13}C NMR** (100 MHz, $CDCl_3$) δ 146.9, 143.0, 131.8, 130.8, 129.8, 125.8, 23.6.

(2-bromo-4-(chlorosulfonyl)phenyl)methylene diacetate

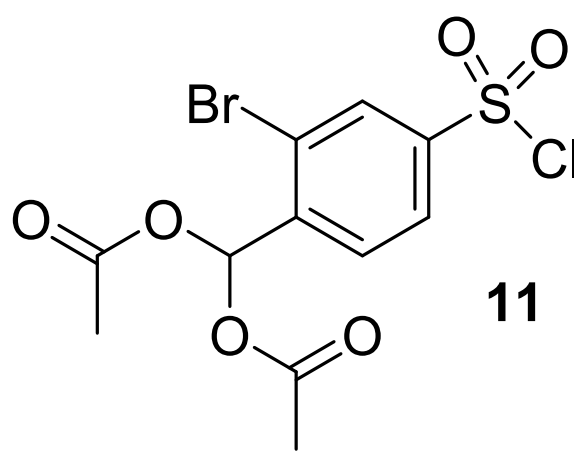

11

A solution of **10** (94.4 g, 350 mmole, 1 eq.) in 500 mL acetic anhydride was cooled to 0°C, and 80 mL 95% sulfuric acid was slowly added. Chromium trioxide (94.5 g, 945 mmole, 2.7 eq.) was slowly added to 435 mL acetic anhydride at 0°C, and the resulting solution was slowly added to the reaction mixture, keeping the internal temperature below 5°C. The reaction was allowed to warm to room temperature and was stirred for 16 h. The reaction was quenched by the addition of isopropyl alcohol. The resulting mixture was diluted with water (5 L, 0°C), stirred for 30 min, and the precipitate was collected by filtration, washed with water (3 times 100 mL) and dried in vacuo to yield (2-bromo-4-(chlorosulfonyl)phenyl)methylene diacetate as a cream solid (31.2 g, 80.9 mmole, 23% yield). **^{1}H NMR** (399 MHz, $CDCl_3$) δ 8.25 (s, 1H), 8.04 (dd, *J* = 8.3, 2.0 Hz, 1H), 7.92 (s, 1H), 7.81 (d, *J* = 8.3 Hz, 1H), 2.17 (s, 6H). **^{13}C NMR** (100 MHz, $CDCl_3$) δ 168.2, 146.0, 142.3, 131.6, 129.5, 126.1, 123.7, 88.1, 20.7.

Focused library compounds 5, 6, 7, 8, 9, 5-OH and 9-OH:

2-bromo-4-((8-hydroxy-3,4-dihydroquinolin-1(2H)-yl)sulfonyl)benzaldehyde (5)

5: 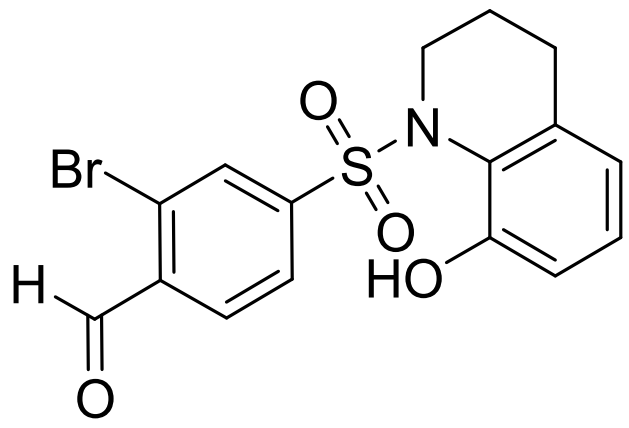

The compound was prepared using the general synthetic procedure, yielding a colorless solid (47.2 mg, 119 µmole, 40% yield). **^{1}H NMR** (400 MHz, Chloroform-*d*) δ 10.36 (s, 1H), 7.93 – 7.86 (m, 2H), 7.58 (dd, *J* = 8.1, 1.8 Hz, 1H), 7.44 (s, 1H), 7.14 (t, *J* = 7.8 Hz, 1H), 6.99 (dd, *J* = 8.3, 1.5 Hz, 1H), 6.60 (dd, *J* = 7.4, 1.4 Hz, 1H), 3.79 (t, *J* = 7.0 Hz, 2H), 1.90 (t, *J* = 7.0 Hz, 2H), 1.80 (q, *J* = 6.7 Hz, 2H) ppm. **^{13}C NMR** (101 MHz, $CDCl_3$) δ 190.4, 152.5, 143.5, 137.3, 136.4, 132.7, 130.4, 129.2, 126.9, 126.7, 124.2, 120.8, 117.3, 47.3, 25.4, 23.1 ppm.

1-((4-formylphenyl)sulfonyl)indoline-2-carboxylic acid (6)

6: 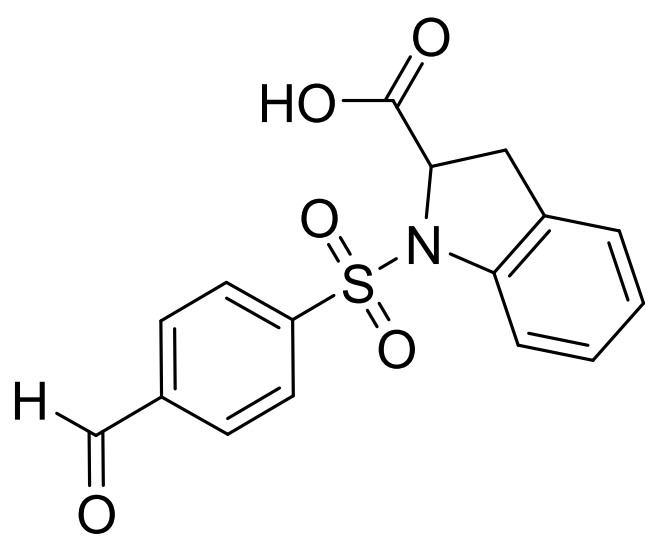

The compound was prepared using the general synthetic procedure, yielding a pink solid (34.8 mg, 105 µmole, 56% yield). **^{1}H NMR** (400 MHz, Chloroform-*d*) δ 10.04 (s, 1H), 8.03 – 7.91 (m, 4H), 7.58 (d, *J* = 8.1 Hz, 1H), 7.29 – 7.20 (m, 1H), 7.05 (td, *J* = 7.3, 1.0 Hz, 2H), 5.35 (s, 2H), 4.86 (dd, *J* = 9.9, 5.3 Hz, 1H), 3.30 – 3.14 (m, 2H) ppm. **^{13}C NMR** (100 MHz, $CDCl_3$) δ 190.9, 175.0, 142.5, 140.6, 139.5, 130.4, 129.6, 128.6, 128.1, 125.4, 125.2, 115.6, 62.2, 33.0 ppm.

2-bromo-4-((4-hydroxy-3,4-dihydroquinolin-1(2H)-yl)sulfonyl)benzaldehyde (7)

7: 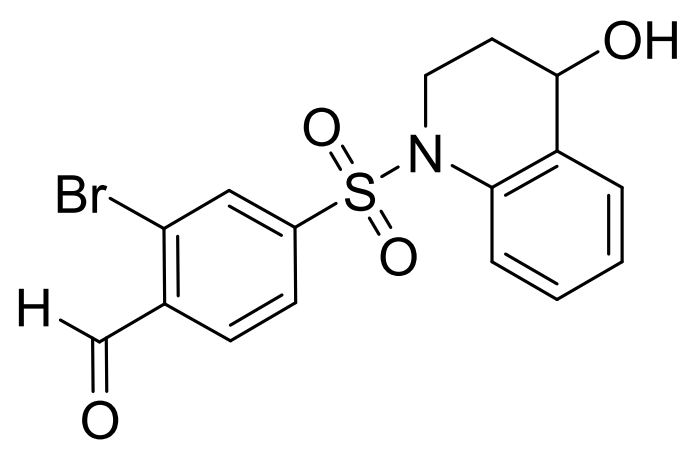

The compound was prepared using the general synthetic procedure, yielding a yellow solid (56.4 mg, 142 µmole, 96% yield). **^{1}H NMR** (399 MHz, Chloroform-*d*) δ 10.33 (d, *J* = 0.8 Hz, 1H), 7.95 (d, *J* = 1.7 Hz, 1H), 7.89 (d, *J* = 8.1 Hz, 1H), 7.82 (dd, *J* = 8.8, 1.2 Hz, 1H), 7.61 (ddd, *J* = 8.1, 1.8, 0.8 Hz, 1H), 7.38 – 7.29 (m, 2H), 7.20 (td, *J* = 7.4, 1.2 Hz, 1H), 4.54 (q, *J* = 4.2 Hz, 1H), 4.19 – 4.07 (m, 1H), 3.81 (ddd, *J* = 13.5, 10.9, 3.7 Hz, 1H), 1.89 (dtd, *J* = 13.4, 4.9, 3.8 Hz, 1H), 1.84 – 1.72 (m, 1H), 1.68 (d, *J* = 4.3 Hz, 1H) ppm. **^{13}C NMR** (100 MHz, $CDCl_3$) δ 190.5, 145.2, 136.2, 135.5, 132.5, 131.5, 130.5, 129.4, 129.3, 127.1, 126.2, 125.9, 123.6, 64.9, 42.8, 30.3 ppm.

4-((3-hydroxy-3,4-dihydroquinolin-1(2H)-yl)sulfonyl)benzaldehyde (8)

8: 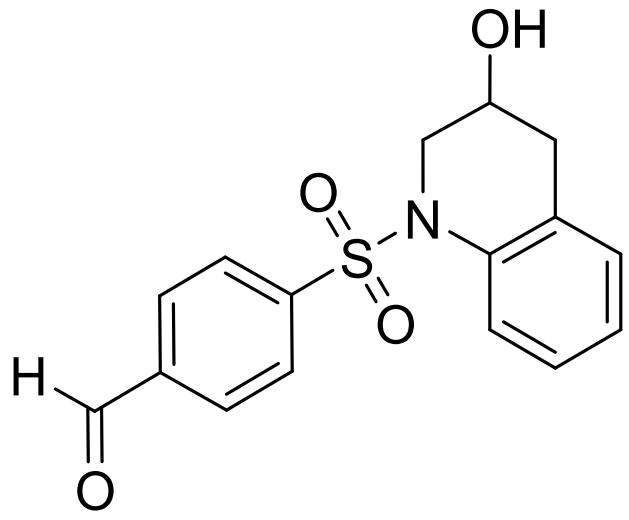

The compound was prepared using the general synthetic procedure, yielding a yellow oil (25.0 mg, 79 µmole, 22% yield). **^{1}H NMR** (400 MHz, Chloroform-*d*) δ 10.02 (t, *J* = 2.7 Hz, 1H), 7.91 (dt, *J* = 8.5, 2.0 Hz, 2H), 7.85 (dd, *J* = 8.4, 2.1 Hz, 2H), 7.71 – 7.63 (m, 1H), 7.20 (td, *J* = 7.7, 2.0 Hz, 1H), 7.10 (ddd, *J* = 9.5, 5.6, 1.8 Hz, 1H), 7.04 (dd, *J* = 7.4, 2.1 Hz, 1H), 4.10 – 3.97 (m, 2H), 3.70 (ddt, *J* = 12.5, 6.1, 3.2 Hz, 1H), 2.77 (dd, *J* = 16.7, 5.1 Hz, 1H), 2.58 – 2.43 (m, 2H) ppm. **^{13}C NMR** (101 MHz, $CDCl_3$) δ 191.1 (m, mixture of rotamers), 144.6 (m, mixture of rotamers), 138.9, 135.7, 130.2, 130.1, 127.9, 127.2 (m, mixture of rotamers), 125.8, 123.4 (m, mixture of rotamers), 64.1 (m, mixture of rotamers), 52.4, 35.8 ppm.

2-bromo-4-((3-hydroxy-3,4-dihydroquinolin-1(2H)-yl)sulfonyl)benzaldehyde (9)

9: 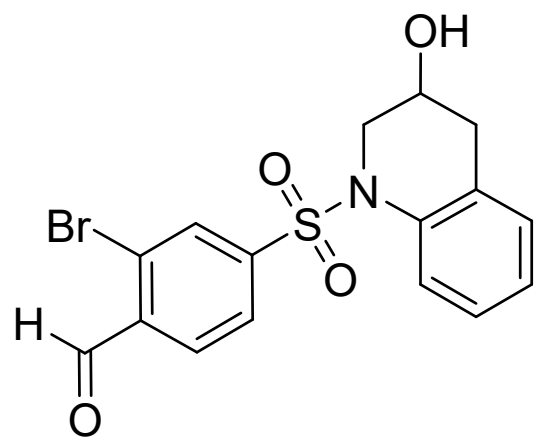

The compound was prepared using the general synthetic procedure, yielding a yellow oil (77.1 mg, 195 µmole, 65% yield). **^{1}H NMR** (400 MHz, Chloroform-*d*) δ 10.33 (s, 1H), 8.01 (d, *J* = 1.7 Hz, 1H), 7.90 (d, *J* = 8.2 Hz, 1H), 7.75 – 7.53 (m, 2H), 7.25 – 7.19 (m, 1H), 7.13 (td, *J* = 7.4, 1.3 Hz, 1H), 7.07 (dd, *J* = 7.4, 1.6 Hz, 1H), 4.17 – 4.09 (m, 1H), 3.97 (ddd, *J* = 13.6, 3.8, 0.9 Hz, 1H), 3.80 (ddd, *J* = 13.6, 6.4, 0.9 Hz, 1H), 2.84 (dd, *J* = 16.5, 5.4 Hz, 1H), 2.54 (dd, *J* = 16.6, 6.1 Hz, 1H), 2.12 (s, 1H) ppm. **^{13}C NMR** (101 MHz, $CDCl_3$) δ 190.6, 145.6, 136.1, 135.5, 132.6, 130.4, 130.2, 127.8, 127.4, 127.0, 126.4, 126.0, 123.4, 64.3, 52.5, 35.9 ppm.

1-((3`-bromo-4`-(hydroxymethyl)phenyl)sulfonyl)-1,2,3,4-tetrahydroquinolin-8-ol (5-OH)

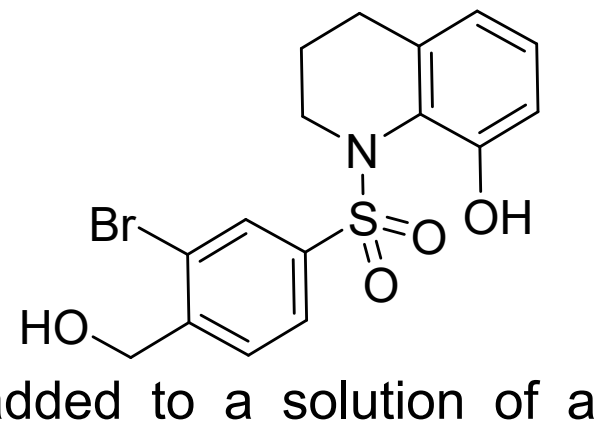

$NaBH_4$ (2.63 mg, 69.4 µmole) was added to a solution of aldehyde **5** (25.0 mg, 63.1 µmole) in THF/MeOH (1 mL, 1:1 v/v). The solution was stirred 2 h at room temperature. Aqueous NaCl solution (sat., 5 mL) was added and the aqueous layer was extracted with EtOAc (4 x 10 mL). The combined organic extracts were treated with Na_2SO_4 and evaporated. Flash column chromatography of the residue (4 g silica, cyclohexane/EtOAc 8% → 80%) afforded primary alcohol **5-OH** as a colorless solid containing 0.33 eq. EtOAc (18.7 mg, 31.5 µmole, 50%). **^{1}H-NMR** (399 MHz, $CDCl_3$): *δ* = 7.75 (d, *J* = 1.6 Hz, 1H), 7.61 – 7.55 (m, 2H), 7.48 (dd, *J* = 8.1, 1.6 Hz, 1H), 7.11 (t, *J* = 7.8 Hz, 1H), 6.96 (d, *J* = 8.0 Hz, 1H), 6.57 (d, *J* = 7.4 Hz, 1H), 4.77 (d, *J* = 5.2 Hz, 2H), 3.75 (t, *J* = 7.0 Hz, 2H), 2.24 (t, *J* = 5.8 Hz, 1H), 1.88 (t, *J* = 7.1 Hz, 2H), 1.75 (q, *J* = 6.9 Hz, 2H) ppm.
^{13}C-NMR (100 MHz, $CDCl_3$): *δ* = 152.6, 145.9, 138.0, 137.3, 131.1, 128.8, 128.3, 126.5, 124.7, 121.9, 120.7, 117.0, 64.3, 47.1, 25.3, 23.1 ppm.

1-((3`-bromo-4`-(hydroxymethyl)phenyl)sulfonyl)-1,2,3,4-tetrahydroquinolin-3-ol (9-OH)

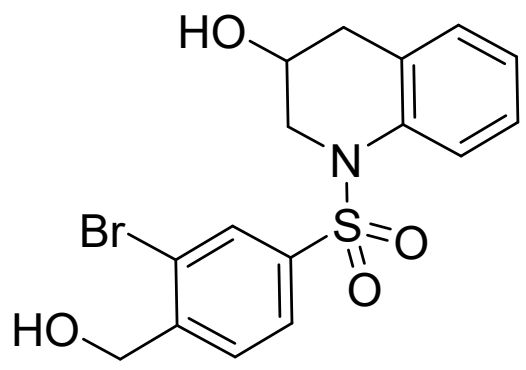

$NaBH_4$ (4.74 mg, 125 µmole) was added to a solution of aldehyde **9** (24.8 mg, 62.6 µmole) in THF/MeOH (1 mL, 1:1 v/v). The solution was stirred 2 h at room temperature. Aqueous NaCl solution (sat., 5 mL) was added and the aqueous layer was extracted with EtOAc (4 x 10 mL). The combined organic extracts were treated with Na_2SO_4 and evaporated. Flash column chromatography of the residue (4 g silica, cyclohexane/EtOAc 8% → 80%) afforded primary alcohol **9-OH** as a colorless solid (17.2 mg, 43.2 µmole, 69%).

^{1}H-NMR (399 MHz, acetone-d$_6$): δ = 7.82 (d, J = 1.9 Hz, 1H), 7.76 – 7.69 (m, 3H), 7.20 (dt, J = 8.5, 4.4 Hz, 1H), 7.09 (d, J = 4.4 Hz, 2H), 4.74 (t, J = 5.6 Hz, 1H), 4.68 (d, J = 5.0 Hz, 2H), 4.38 (d, J = 4.3 Hz, 1H), 4.12 (ddd, J = 13.1, 4.2, 1.1 Hz, 1H), 3.97 (dq, J = 7.9, 3.7 Hz, 1H), 3.55 (dd, J = 13.3, 7.8 Hz, 1H), 2.79 (dd, J = 16.3, 5.4 Hz, 1H), 2.47 (dd, J = 16.2, 7.5 Hz, 1H) ppm.

^{13}C-NMR (100 MHz, acetone-d$_6$): δ = 147.6, 140.5, 136.9, 131.3, 130.8, 129.7, 129.0, 127.4, 127.2, 125.9, 123.9, 121.8, 63.9, 63.92, 53.2, 36.7 ppm.

Supporting Figures and Tables

SI Table S1. Focused library profiling potency and stabilization factors for the stabilization of the 14-3-3/Tau pS214 pS324 (pS2) interaction.

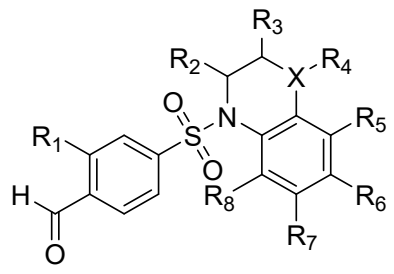

series 1

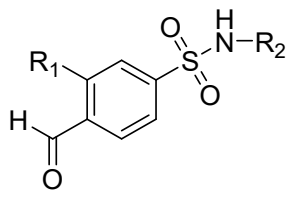

series 2

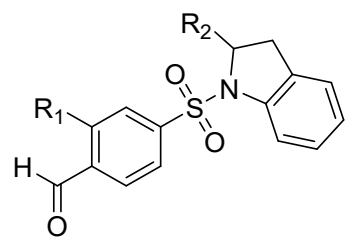

series 3

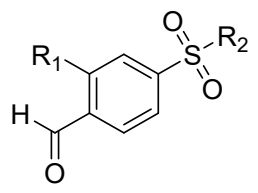

series 4

compound	R_1	R_2	R_3	R_4	R_5	R_6	R_7	R_8	X	fold increase	CC_{50} (µM)	apparent $K_{D,250}$ (nM)	SF
**series 1**													
**FM29**	H	H	H	Me	H	H	H	H	C	1.45 ± 0.01	-		-
**FM38**	H	H	H	H	H	H	Br	H	C	1.30 ± 0.01	-		-
**FM41**	H	H	H	H	H	OMe	H	H	C	1.01 ± 0.02	-		-
**FM43**	H	H	H	OH	H	H	H	H	C	1.51 ± 0.03	-		-
**FM50**	H	H	H	H	H	H	Cl	H	C	1.01 ± 0.01	-		-
**FM55**	Br	H	H	Me	H	H	H	H	C	1.23 ± 0.01	-		-
**7**	Br	H	H	OH	H	H	H	H	C	2.30 ± 0	160.5 ± 10.3	102.4 ± 17.0	13.4 ± 5.1
**FM57**	Br	H	H	H	H	H	Br	H	C	1.78 ± 0.03	-		-
**FM59**	Br	H	H	H	H	OMe	H	H	C	0.95 ± 0.01	-		-
**FM62**	H	H	H	H	H	H	H	OH	C	1.78 ± 0.03	-		-
**FM63**	H	H	H	H	H	H	H	Me	C	1.10 ± 0	-		-
**FM66**	H	Me	H	H	H	H	H	H	C	1.07 ± 0.01	-		-
**8**	H	H	OH	H	H	H	H	H	C	2.56 ± 0	108.1 ± 0.4	85.7 ± 13.9	15.5 ± 4.7
**FM69**	H	H	H	H	H	methyl acetate	H	H	C	1.11 ± 0.03	-		-
**5**	Br	H	H	H	H	H	H	OH	C	2.49 ± 0.02	94.8 ± 12.7	27.1 ± 5.4	46.8 ± 1.8
**FM78**	Br	H	H	H	H	H	H	Me	C	1.04 ± 0.02	-		-
**FM79**	Br	Me	H	H	H	H	H	H	C	1.01 ± 0	-		-
**FM85**	Br	H	Me	H	H	H	H	H	C	1.25 ± 0.02	-		-
**9**	Br	H	OH	H	H	H	H	H	C	2.06 ± 0.02	44.3 ± 8.0	19.8 ± 4.5	53.1 ± 10.2
**FM95**	Br	H	H	H	F	H	H	H	O	1.34 ± 0.01	-		-
**FM101**	Br	H	H	H	Me	H	H	H	C	1.20 ± 0.02	-		-
**FM102**	Br	H	H	H	OMe	H	H	H	C	1.17 ± 0.02	-		-
**FM105**	Br	H	H	geminal dimethyl	H	H	H	H	C	1.38 ± 0.02	-		-
**FM106**	Br	H	H	Cl	H	H	H	H	C	1.05 ± 0.01	-		-
**FM108**	Br	H	H	=O	H	H	H	H	C	1.20 ± 0.01	-		-
**FM110**	Br	H	H	H	Cl	H	H	H	O	1.36 ± 0.02	-		-
**AO57**	Br	H	H	H	H	H	OH	H	C	1.78 ± 0.03	-		-
**AO58**	Br	H	H	Me	H	H	H	H	N	1.45 ± 0.07	-		-
**AO59**	Br	H	=O	H	H	H	H	H	N	1.45 ± 0.01	-		-
**AO68**	Br	H	H	-	H	H	F	H	O	1.34 ± 0.01	-		-
**AO82**	Br	H	H	isopropyl	H	H	H	H	N	1.12 ± 0.03	-		-
**AO55**	Br	H	H	Me	H	H	H	OH	C	1.27 ± 0.01	-		-
**AO56**	Br	H	H	acetamide	H	H	H	H	C	1.72 ± 0.01	-		-
**AO77**	Br	H	OH	H	H	H	H	OH	C	1.14 ± 0	-		-

series 2						
**compound**	**R_1**	**R_2**	**fold increase**	**CC_{50} (μM)**	**apparent $K_{D,250}$ (nM)**	**SF**
**FM3**	H	2,3-dihydro-1*H*-inden-1-yl	1.22 ± 0.01			
**FM5**	H	adamantan-1-yl	1.34 ± 0.01			
**FM7**	H	9*H*-fluoren-2-yl	1.05 ± 0.02			
**FM10**	H	cyclopropyl	1.33 ± 0.02			
**FM11**	H	cyclobutyl	1.38 ± 0.01			
**FM14**	Br	cyclopropyl	1.87 ± 0.03			
**FM15**	Br	cyclobutyl	1.90 ± 0.01			
**FM17**	Br	adamantan-1-yl	1.35 ± 0			
**FM20**	Br	9*H*-fluoren-2-yl	1.32 ± 0.02			
**AO79**	Br	(*S*) 2,3-dihydro-1*H*-inden-1-yl	1.63 ± 0			
**AO78**	Br	(*R*) 2,3-dihydro-1*H*-inden-1-yl	1.19 ± 0.01			

series 3						
**compound**	**R_1**	**R_2**	**fold increase**	**CC_{50} (μM)**	**apparent $K_{D,250}$ (nM)**	**SF**
**6**	H	COOH	2.21 ± 0.01	248.9 ± 19.3	269.1 ± 38.6	5.2 ± 1.9
**FM49**	H	Me	1.64 ± 0			-
**FM87**	Br	Me	1.05 ± 0.03			-
**FM91**	Br	H	1.09 ± 0			-
**series 4**						
**FM39**	H	2,3,4,9-tetrahydro-1*H*-pyrido[3,4-b]indole	0.96 ± 0.03			
**FM48**	H	1-cyclohexyl piperazine	1.05 ± 0			
**FM48F2**	H	1-cyclohexyl piperazine	1.10 ± 0			
**FM74**	Br	1-cyclohexyl piperazine	1.26 ± 0.02			
**FM96**	Br	7-azaspiro[3.5]nonane	1.19 ± 0.03			
**FM98**	Br	6-azaspiro[2.5]octane	1.24 ± 0.03			

SI Table S2. Data collection and refinement statistics of crystal structures

**PDB**	**9GG7**	**9GG8**	**9GGA**
Protein	14-3-3σΔc	14-3-3σΔc	14-3-3σΔc
Peptide	Tau pS324	Tau pS214	Tau pS214
Compound	**1**	1	**9**
Beam	MASSIF-1	MASSIF-1	ID30B
Data collection			
Wavelength (Å)	0.965459	0.965459	0.885601
Space group	P 1 2 1 1	C 2 2 21	C 2 2 21
Cell dimensions			
a, b, c (Å)	69.2, 62.5, 69.3	82.3, 112.5, 62.5	82.5, 112.3, 62.7
α, β, γ (°)	90.0, 107.67, 90.0	90.0, 90.0, 90.0	90.0, 90.0, 90.0
Resolution (Å)	65.97-1.24	66.40-1.10	66.50-2.24
	(1.26-1.24)	(1.12-1.10)	(2.32-2.24)
I / σ(I)	7.7 (1.3)	13.7 (1.72)	18.5 (6.0)
Completeness (%)	97.5 (93.6)	99.9 (99.5)	99.0 (99.5)
Redundancy	4.4 (4.3)	6.3 (6.3)	5.1 (5.0)
$CC_{1/2}$	0.981 (0.414)	0.990 (0.719)	0.995 (0.841)
Refinement			
No. reflections	347914	746247	72005
R_{work}/R_{free}	0.220 / 0.242	0.199 / 0.203	0.194 / 0.199
No. atoms			
Overall	3833	1948	1872
Solvent	398	224	60
B-factors			
Overall	18.07	14.42	30.09
R.m.s. deviations			
Bond lengths (Å)	0.013	0.014	0.0151
Bond angles (°)	1.751	1.652	1.860
Ramachandran			
favored (%)	98.09	98.32	99.56
outliers (%)	0.00	0.00	0.00

SI Table S3. Smiles strings of the focused library of 67 aldehydes

Compound identity	Smiles strings
TCF000521-108	OCC1=CN(C=N1)C1=NC=C(C=O)C=C1
TCF000521-109	NC(=O)C1=CN(C=N1)C1=NC=C(C=O)C=C1
TCF000521-112	ClC1=CN(C=N1)C1=NC=C(C=O)C=C1
TCF000521-113	CC(=O)C1=CN(C=N1)C1=NC=C(C=O)C=C1
TCF000521-114	CC(=O)NC1=CC=C(O)C=C1
TCF000521-115	COC1=CC=C(NC(C)=O)C=C1
TCF000521-116	COC1=CC=C2N(C(Cl)=NC2=C1)S(=O)(=O)C1=CC=C(C=O)C=C1
TCF000521-117	CN1CCCN(CC1)S(=O)(=O)C1=CC=C(C=O)C=C1
TCF000521-118	CN1CCN(CC1)S(=O)(=O)C1=CC=C(C=O)C=C1
TCF000521-119	CN(C1CCCCC1)S(=O)(=O)C1=CC=C(C=O)C=C1
TCF000521-120	CN(C1CCOCC1)S(=O)(=O)C1=CC=C(C=O)C=C1
TCF000521-121	COC1CCCN(C1)S(=O)(=O)C1=CC=C(C=O)C=C1
TCF000521-122	COC1CCN(CC1)S(=O)(=O)C1=CC=C(C=O)C=C1
TCF000521-123	CC(=O)N1CCN(CC1)S(=O)(=O)C1=CC=C(C=O)C=C1
TCF000521-124	CC(C)CN1CCN(CC1)S(=O)(=O)C1=CC=C(C=O)C=C1
TCF000521-125	COCCN1CCN(CC1)S(=O)(=O)C1=CC=C(C=O)C=C1
TCF000521-126	O=CC1=CC=C(C=C1)S(=O)(=O)N1CCC(CC1)N1CCCC1
TCF000521-127	C[C@H]1CCCN1S(=O)(=O)C1=CC=C(C=O)C=C1
TCF000521-128	O=CC1=CC=C(C=C1)S(=O)(=O)N1CCc2ccccc2C1
TCF000521-129	CC1CN(CC(C)O1)S(=O)(=O)C1=CC=C(C=O)C=C1
TCF000521-130	O=CC1=CC=C(C=C1)S(=O)(=O)N1CCNC(=O)C1
TCF000521-131	OC1CCCN(C1)S(=O)(=O)C1=CC=C(C=O)C=C1
TCF000521-132	O=CC1=CC=C(C=C1)S(=O)(=O)N1CCCCC1
TCF000521-133	O=CC1=CC=C(C=C1)S(=O)(=O)N1CCCc2ccccc12
TCF000521-134	CC(C)N1CCN(CC1)S(=O)(=O)C1=CC=C(C=O)C=C1
TCF000521-135	CN(C)C1CCN(CC1)S(=O)(=O)C1=CC=C(C=O)C=C1
TCF000521-158	COC1CN(C1)C(=O)C1=CC=C(C=O)C=C1
TCF000521-159	CN1C=CC(NC(=O)C2=CC=C(C=O)C=C2)=N1
TCF000521-160	COC1=CC=C(CNC(=O)C2=CC=C(C=O)C=C2)C=C1
TCF000521-161	O=CC1=CC=C(C=C1)C(=O)N1CCOCC1
TCF000521-162	CCN1CCN(CC1)C(=O)C1=CC=C(C=O)C=C1
TCF000521-163	COC1=CC(C)=C(NC(=O)C2=CC=C(C=O)C=C2)C=N1
TCF000521-164	CN(C)C(=O)N1CCN(CC1)C(=O)C1=CC=C(C=O)C=C1
TCF000521-165	CN1C=C(CNC(=O)C2=CC=C(C=O)C=C2)C=N1
TCF000521-166	O=CC1=CC=C(C=C1)C(=O)NCC1CCOCC1
TCF000521-167	COC1=CC=CC(CNC(=O)C2=CC=C(C=O)C=C2)=C1
TCF000521-168	CN1C=CN=C1CNC(=O)C1=CC=C(C=O)C=C1
TCF000521-169	CC(C)N1CCC(CC1)NC(=O)C1=CC=C(C=O)C=C1
TCF000521-170	CN(C)C(=O)CNC(=O)C1=CC=C(C=O)C=C1
TCF000521-171	CN1CCN(CC1)C(=O)C1=CC=C(C=O)C=C1
TCF000521-172	CN(C1CCOCC1)C(=O)C1=CC=C(C=O)C=C1
TCF000521-173	COC1CCN(CC1)C(=O)C1=CC=C(C=O)C=C1
TCF000521-174	COCCN1CCN(CC1)C(=O)C1=CC=C(C=O)C=C1
TCF000521-175	O=CC1=CC=C(C=C1)C(=O)N1CCC(CC1)C#N
TCF000521-176	OC1CCCN(C1)C(=O)C1=CC=C(C=O)C=C1

TCF000521-177	OC1CN(C1)C(=O)C1=CC=C(C=O)C=C1
TCF000521-178	OCCC1CCN(CC1)C(=O)C1=CC=C(C=O)C=C1
TCF000521-179	COC1=CC=C2N(CCCC2=C1)S(=O)(=O)C1=CC=C(C=O)C=C1
TCF000521-180	CC1CCC2=CC=CC=C2N1S(=O)(=O)C1=CC=C(C=O)C=C1
TCF000521-181	FC1=CC=C2N(CCCC2=C1)S(=O)(=O)C1=CC=C(C=O)C=C1
4	ClC1=CC=C2N(CCCC2=C1)S(=O)(=O)C1=CC=C(C=O)C=C1
3	O=CC1=CC=C(C=C1)S(=O)(=O)N1CCOC2=CC=CC=C12
TCF000521-184	FC1=CC=C2OCCN(C2=C1)S(=O)(=O)C1=CC=C(C=O)C=C1
TCF000521-185	COC1=CC=C2N(CCOC2=C1)S(=O)(=O)C1=CC=C(C=O)C=C1
2	ClC1=CC=C2N(CCOC2=C1)S(=O)(=O)C1=CC=C(C=O)C=C1
1	O=CC1=CC=C(C=C1)S(=O)(=O)N1CCNC2=CC=CC=C12
TCF000521-188	O=CC1=CC=C(C=C1)S(=O)(=O)N1CCC2=C1C=CC=C2
TCF000521-189	O=CC1=CC=C(N=C1)N1C=NC2=C1C=CC=C2
TCF000521-190	FC1=CC2=C(C=C1)N=CN2C1=CC=C(C=O)C=N1
TCF000521-191	ClC1=CC2=C(C=C1)N(C=N2)C1=CC=C(C=O)C=N1
TCF000521-192	COC1=CC2=C(C=C1)N(C(C)=N2)C1=CC=C(C=O)C=N1
TCF000521-193	O=CC1=CC=C(N=C1)N1C=NC=C1[C@H]1CCCN1CC1CC1
TCF000521-194	O=CC1=CN=C(C=C1)N1C=NC2=CN=CC=C12
TCF000521-195	O=CC1=CC=CC=C1S(=O)(=O)N1CCCCC1
TCF000521-196	OC1CCN(CC1)S(=O)(=O)C1=CC=CC=C1C=O
TCF000521-197	OC1NCCOS(=O)(=O)C2=C1C=CC=C2
TCF000521-198	OC1NCCCOS(=O)(=O)C2=CC=CC=C12
TCF000521-199	O=CC1=CC=CC=C1C(=O)N1CCCCC1
TCF000521-200	OC1CCN(CC1)C(=O)C1=CC=CC=C1C=O

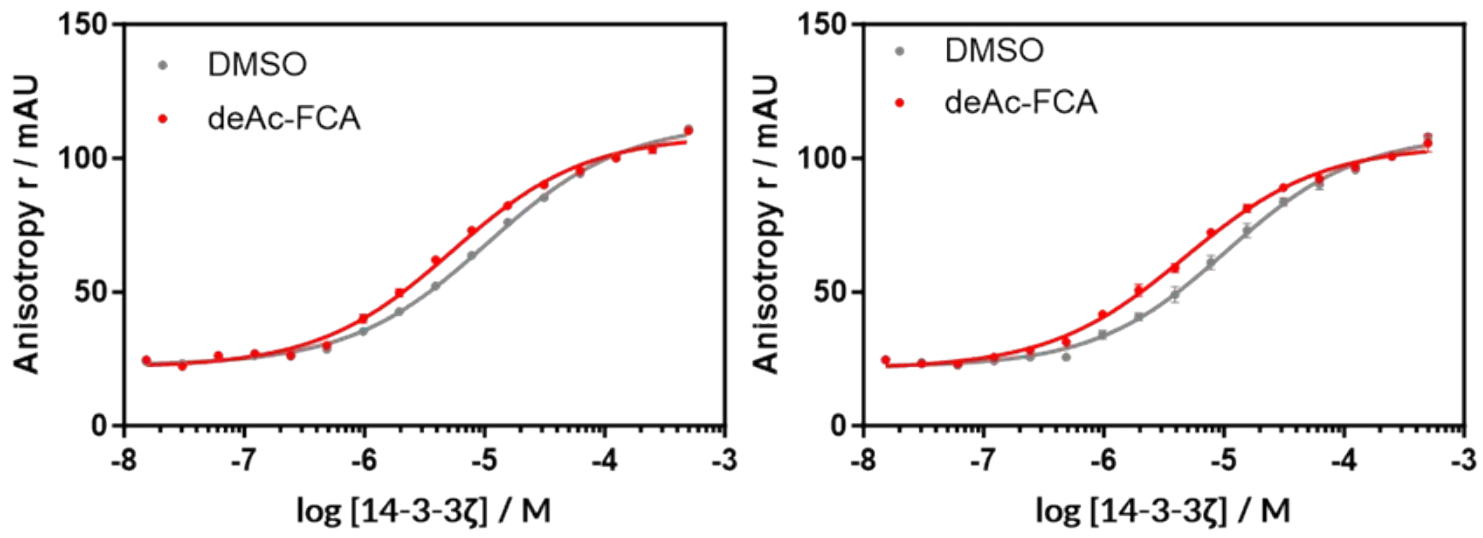

SI Figure S1. Dose-response fluorescence anisotropy data of 14-3-3ζ titrations to 10 nM FITC-labeled pS2 peptide and 100 µM deAc-FCA. Data shown as mean±SD of technical duplicates, with n=2 independent replicates (left & right).

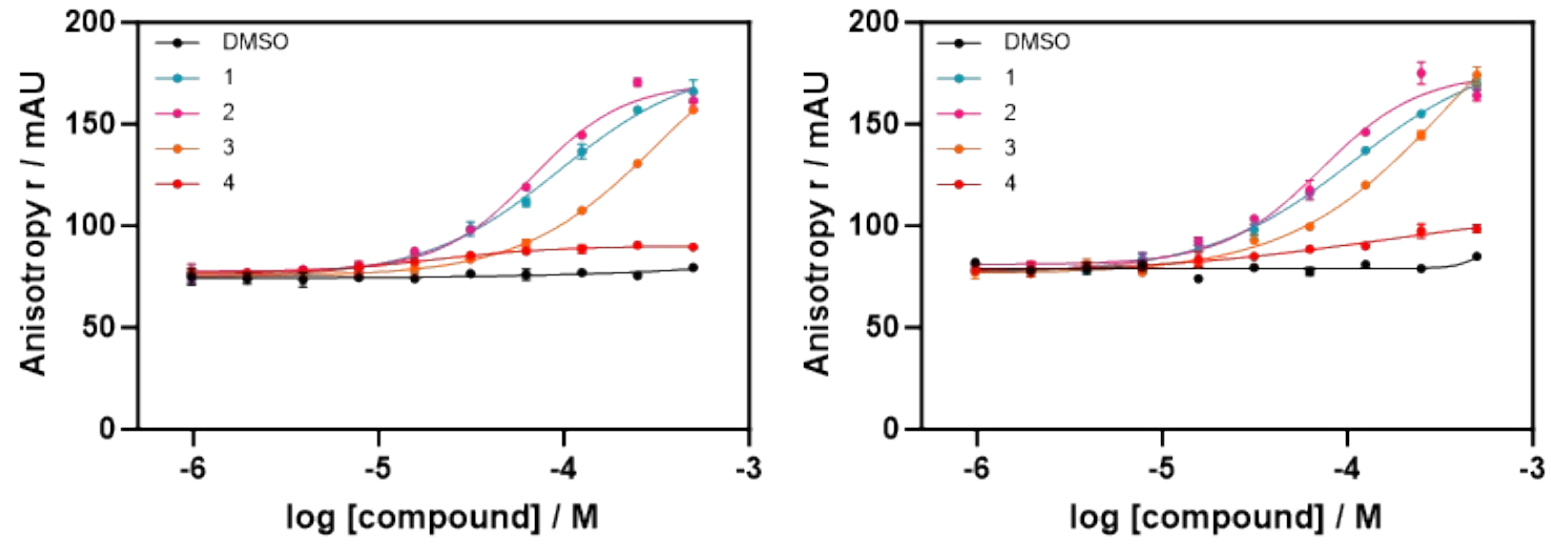

SI Figure S2. Dose-response fluorescence anisotropy data of compound **1-4** titrations and DMSO (black) as negative control to 10 nM FITC-labeled pS2 peptide and 100 nM 14-3-3ζ. Data shown as mean±SD of technical duplicates, with n=2 independent replicates (left & right).

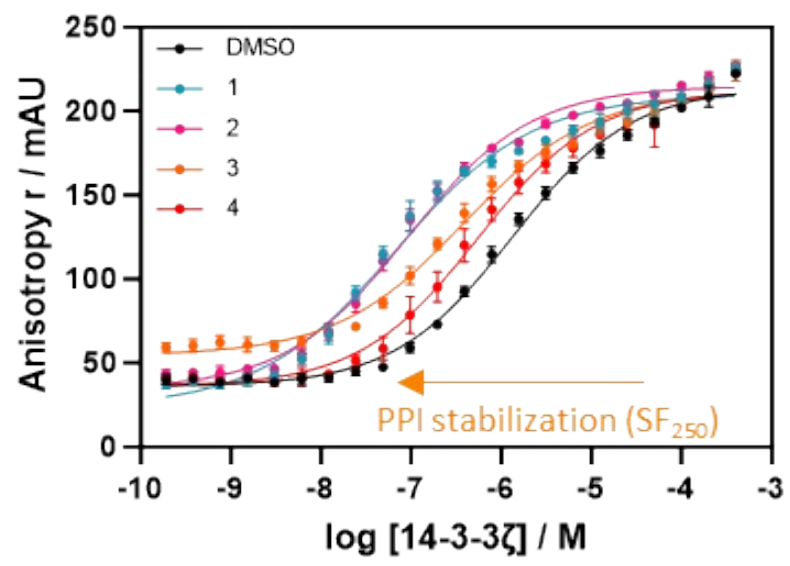

SI Figure S3. Dose-response fluorescence anisotropy data of 14-3-3ζ titrations to 10 nM FITC-labeled pS2 peptide and 250 µM compound. Data shown as mean±SD of n=3 independent replicates.

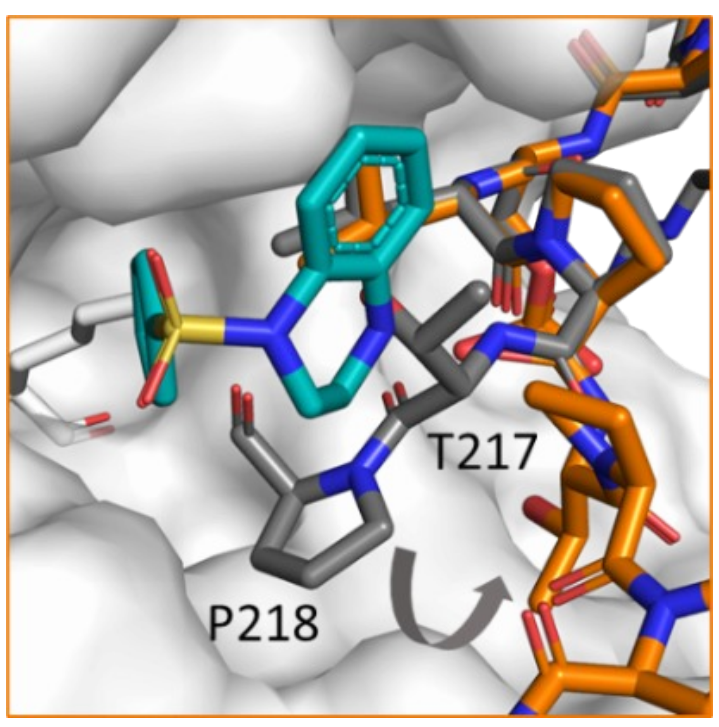

SI Figure S4. Crystal structure of the ternary complex of 14-3-3σΔC (white surface)/Tau pS214 (orange sticks)/compound **1** (blue sticks) (PDB ID: 9GG8), demonstrating the conformational change of residues T217 and P218 compared to the binary crystal structure (grey sticks) (PDB ID: 4FL5).

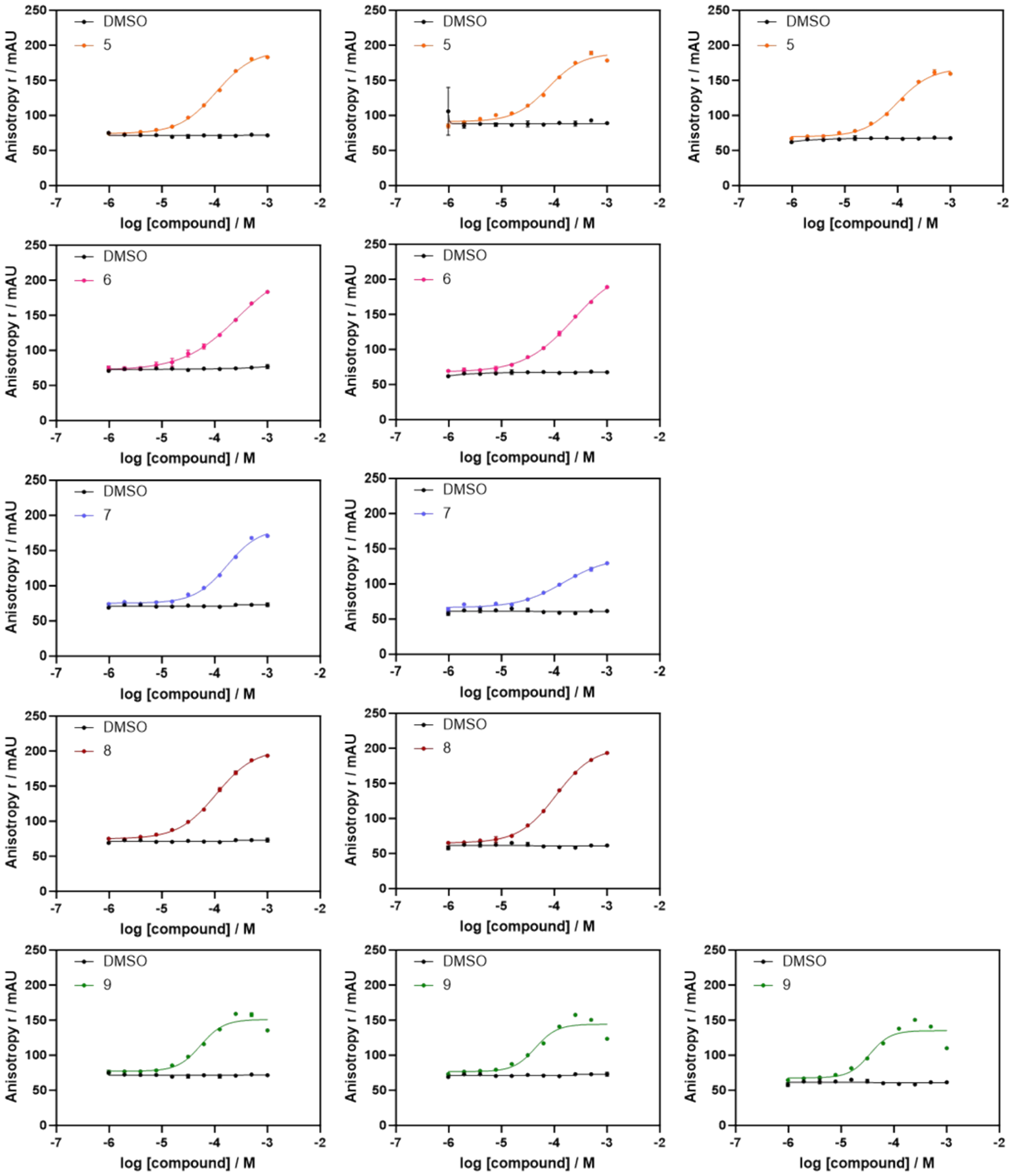

SI Figure S5. Dose-response fluorescence anisotropy data of compound **5-9** titrations and DMSO (black) as negative control to 10 nM FITC-labeled pS2 peptide and 100 nM 14-3-3ζ. Data shown as mean±SD of technical duplicates, with n=2/3 independent replicates.

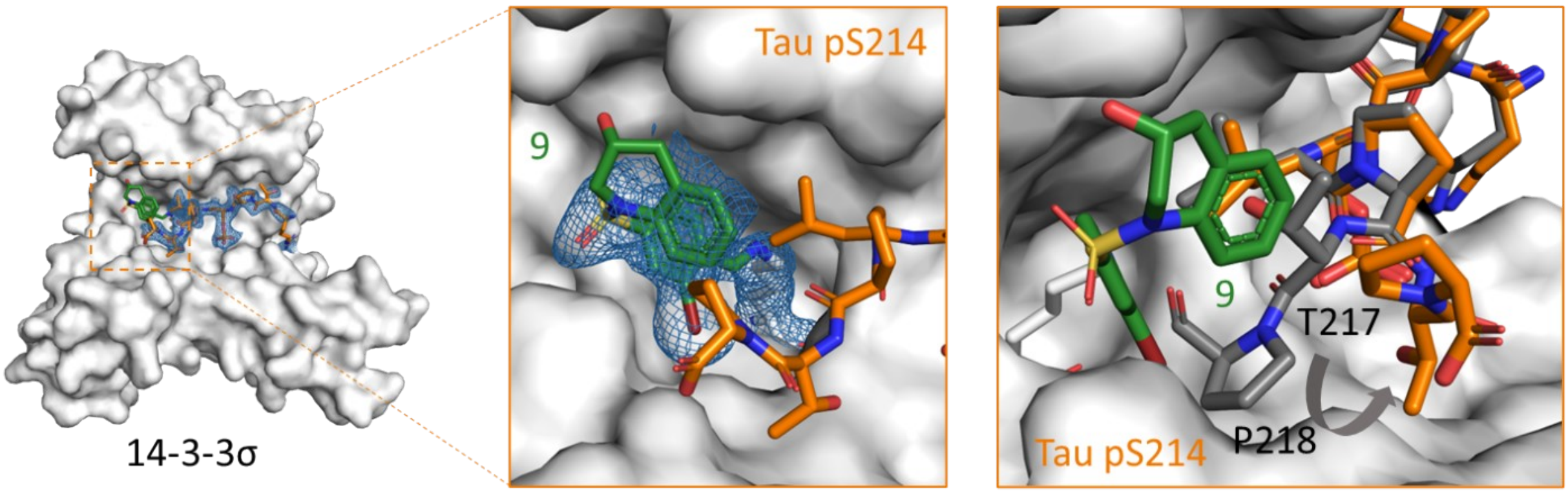

SI Figure S6. Left, front view of a 14-3-3σΔC monomer (white surface) bound by Tau pS214 phosphopeptide (orange sticks) with compound **9** (green sticks). Middle, close-up view of **9** in the 14-3-3 binding groove. 2Fo-Fc electron density maps are contoured at 1σ. Right, conformational change of the Tau pS214 peptide in the binary complex (grey sticks) compared to the tertiary complex (orange sticks). PDB ID: 9GGA.

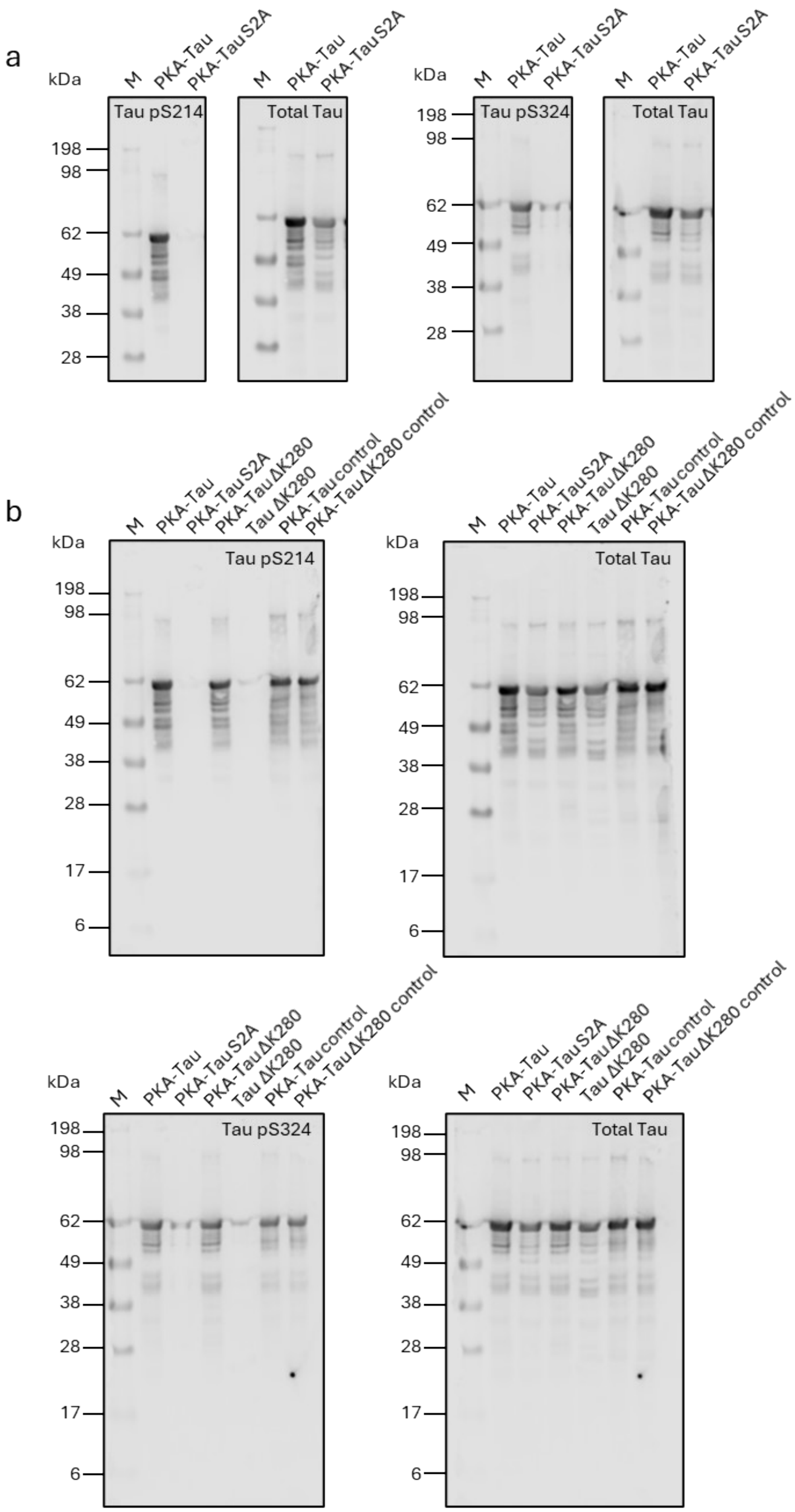

SI Figure S7. a, Western blots of recombinant wildtype and S2A Tau in vitro phosphorylated using PKA kinase using antibodies specific for phosphorylated residues in Tau (Tau pS214 and Tau pS324) and total Tau. b, Full western blots of the recombinant Tau constructs.

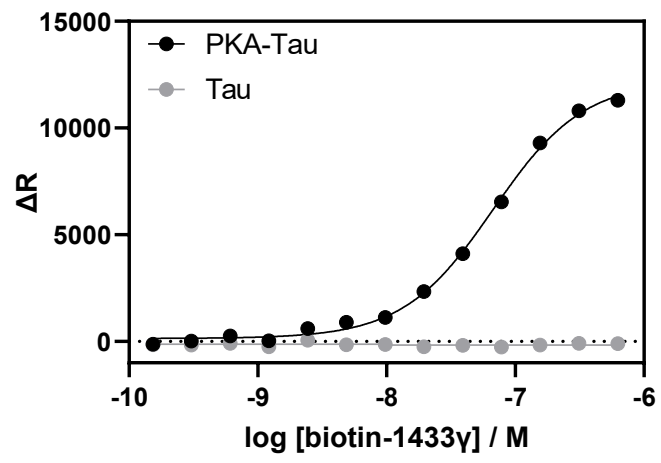

SI Figure S8. ΔR of the TR-FRET dose-response curves of biotin-14-3-3γ and d2-strep (1:4, d2-strep:biotin-14-3-3γ monomer) titration to 40 nM HS-PKA-Tau and HS-Tau. The concentration of anti-His Tb was fixed at 0.5 nM. Data represents n=1

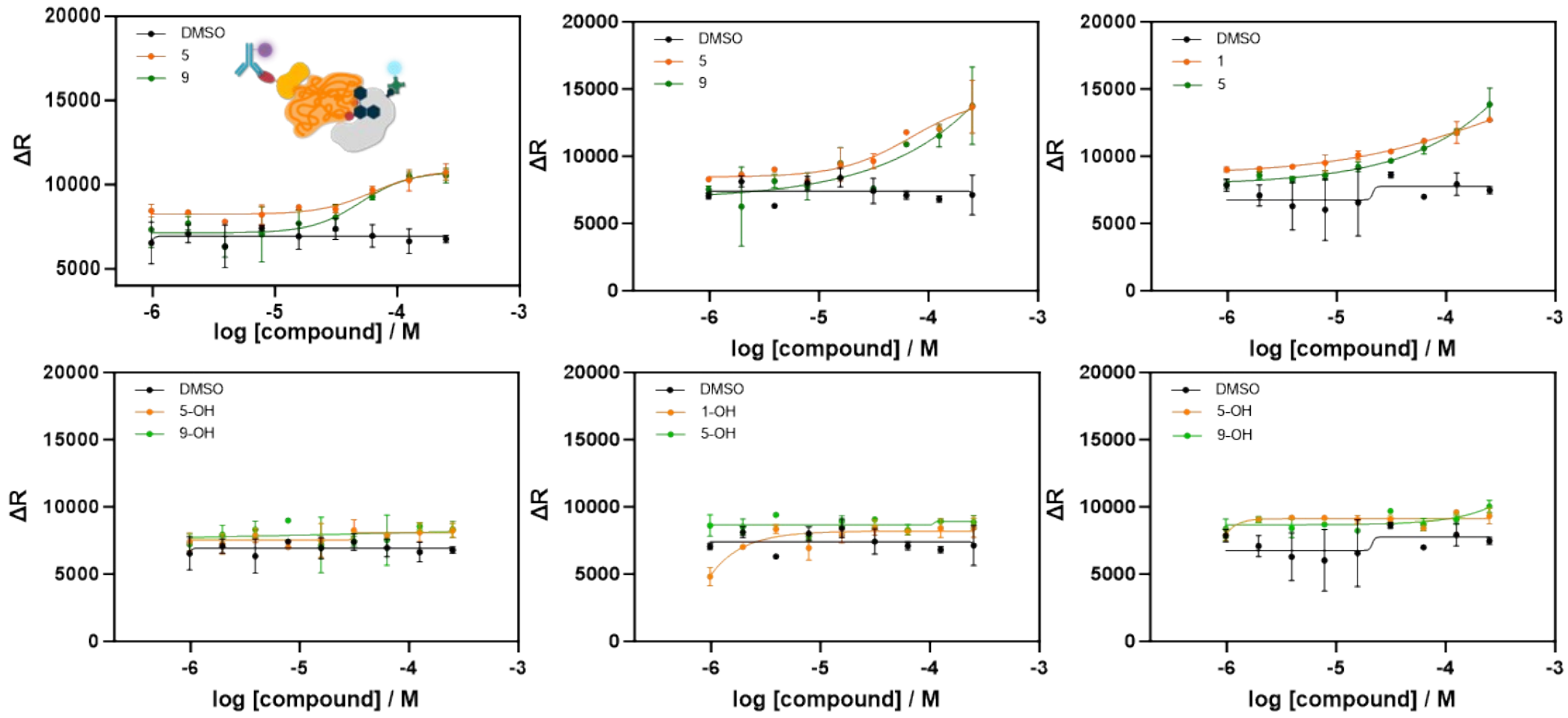

SI Figure S9. ΔR of the TR-FRET dose-response curves of compound titrations to 150 nM biotin-14-3-3γ labeled with d2-strep (1:4, d2-strep:biotin-14-3-3γ monomer) and 40 nM HS-PKA-Tau. The concentration of anti-His Tb was fixed at 0.5 nM. Titration in presence of DMSO was included as a reference. Data and error bars represent mean ± SD of a technical duplicate, three independently performed experiments are shown.

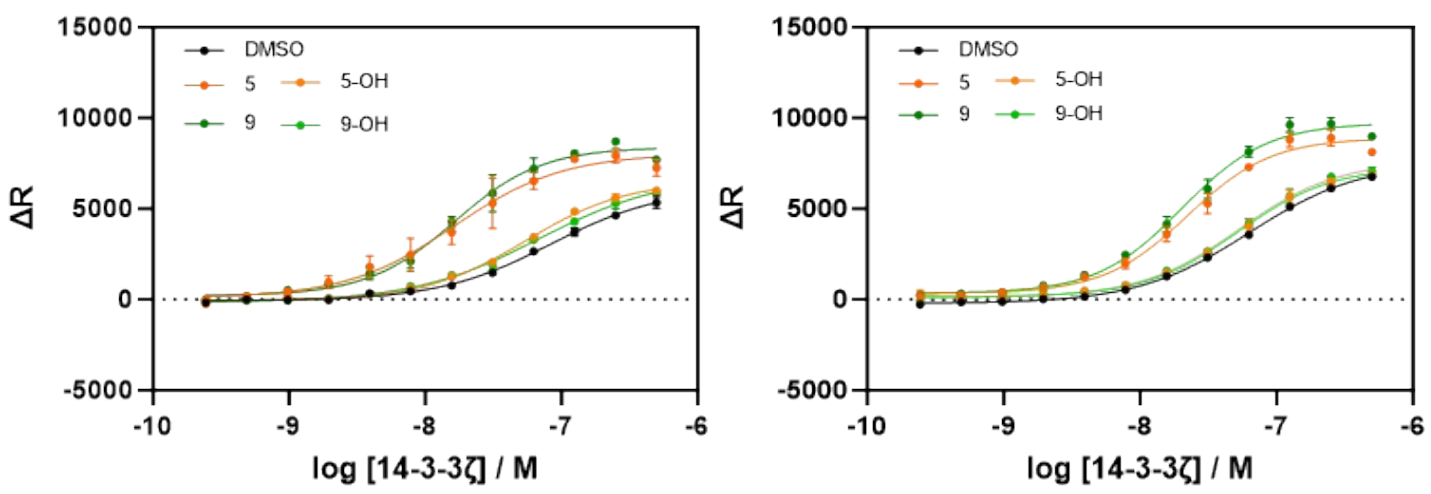

SI Figure S10. ΔR of the TR-FRET dose-response curves of biotin-14-3-3γ and d2-strep (1:4, d2-strep:biotin-14-3-3γ monomer) titration to 40 nM PKA-Tau in presence of DMSO or 250 µM compound. The concentration of anti-His Tb was fixed at 0.5 nM. Data shown representative experiment with n=1, two independently performed experiments.

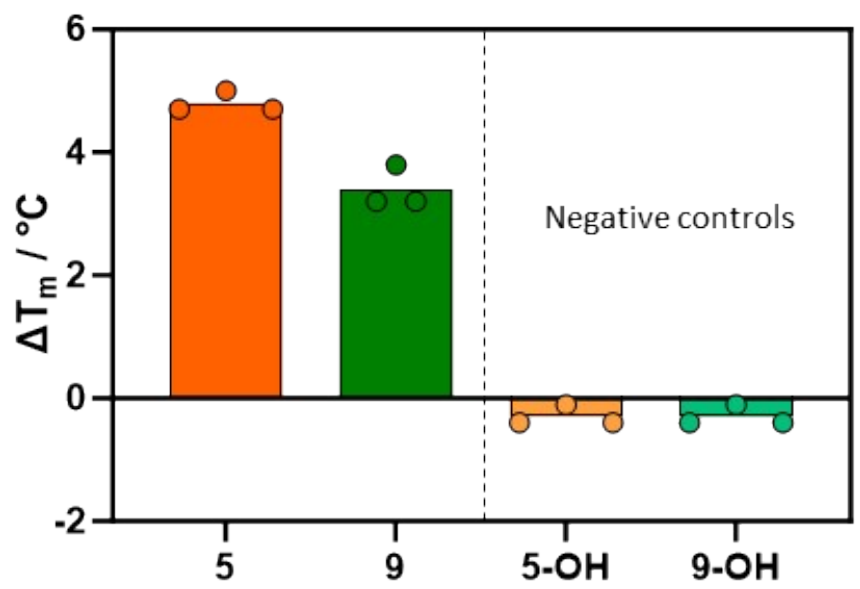

SI Figure S11. DSF assay of PKA-phosphorylated Tau variants binding to 14-3-3ζ in the presence of 250 µM compound. The delta melting temperature ΔT_m is shown, representing the temperature difference compared to the binary 14-3-3ζ/PKA-Tau complex. Data shown as mean, n=3 scientific replicates.

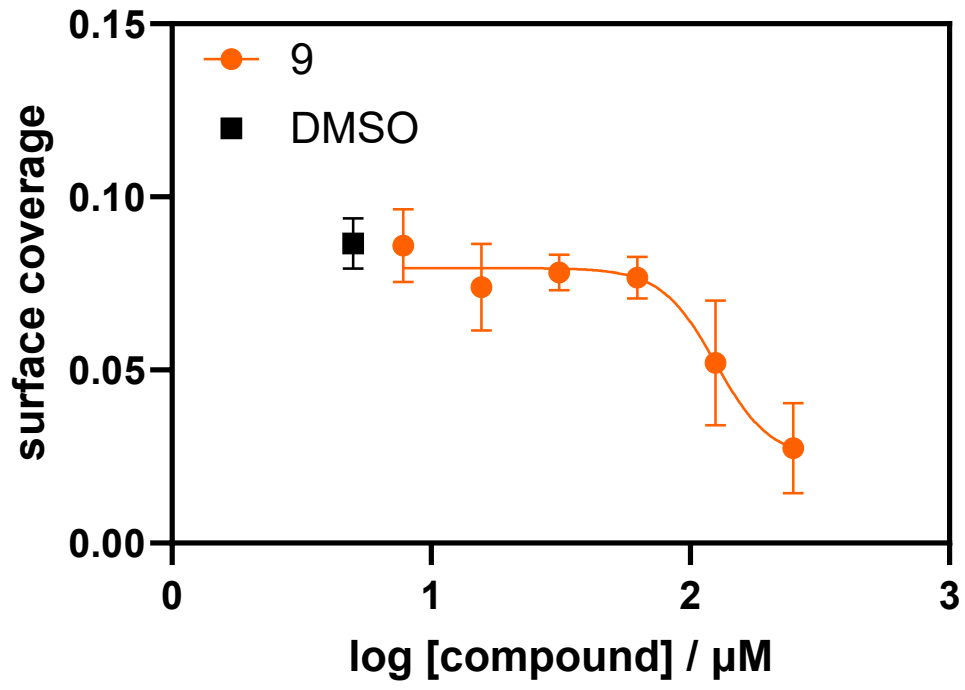

SI Figure S12. Dose-response curve of the surface coverage plotted against the compound **9** concentrations. Data points represent the mean ± SD of 3 independent replicates derived from 3 individual images per data point and condition.

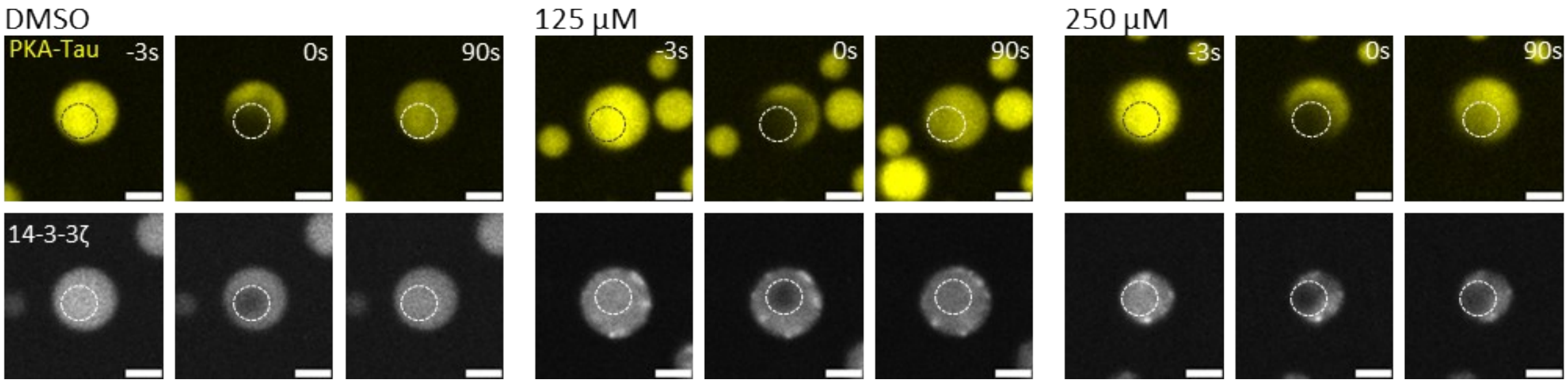

SI Figure S13. Representative FRAP images of PKA-Tau (yellow), (10 µM, 1% DyLight561-labeled Tau) with 14-3-3ζ (white) (7.5 µM, 1% DyLight405-labeled 14-3-3ζ). ROIs are shown (dashed circles). Scale bars of representative FRAP images = 2 µm.

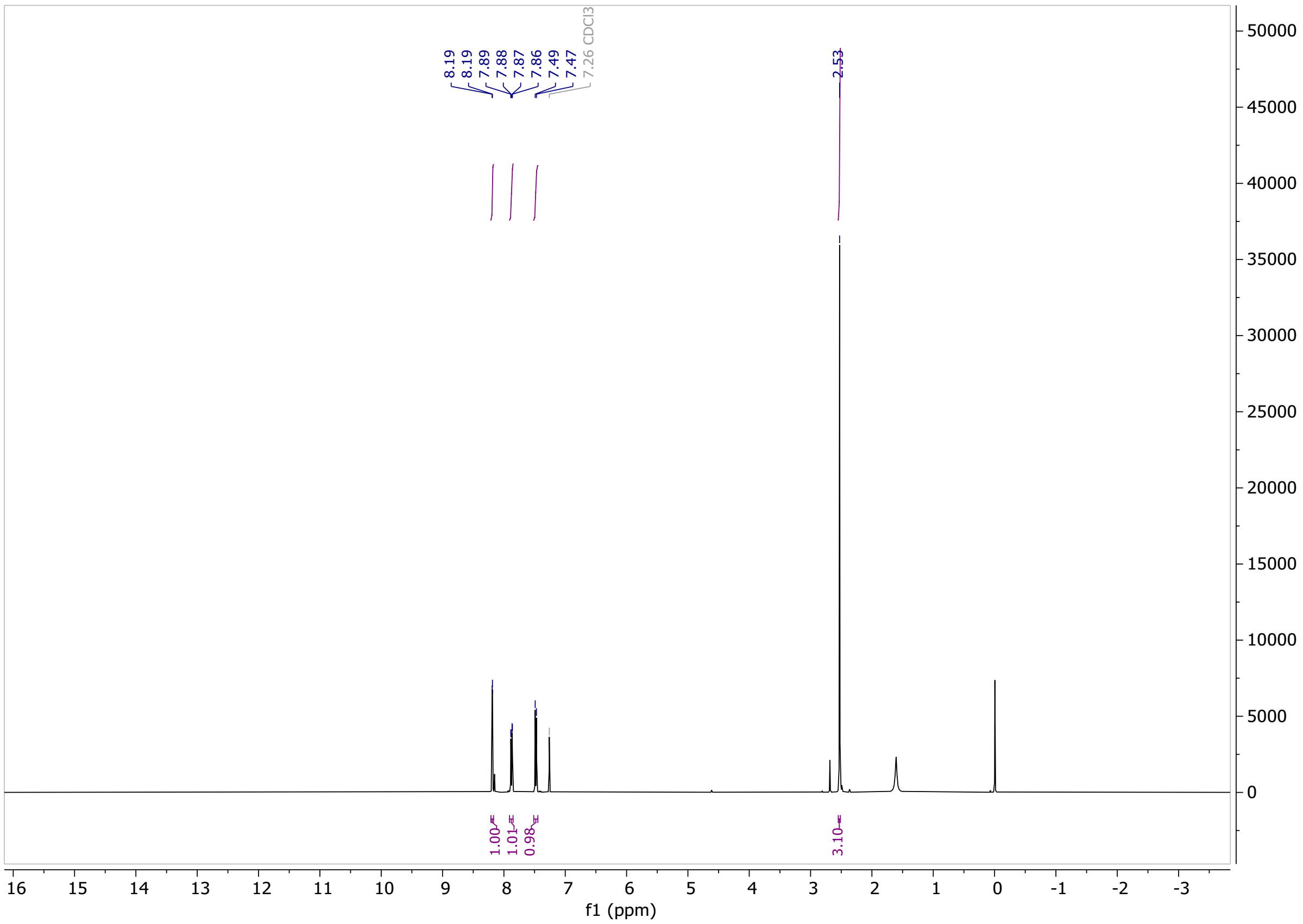

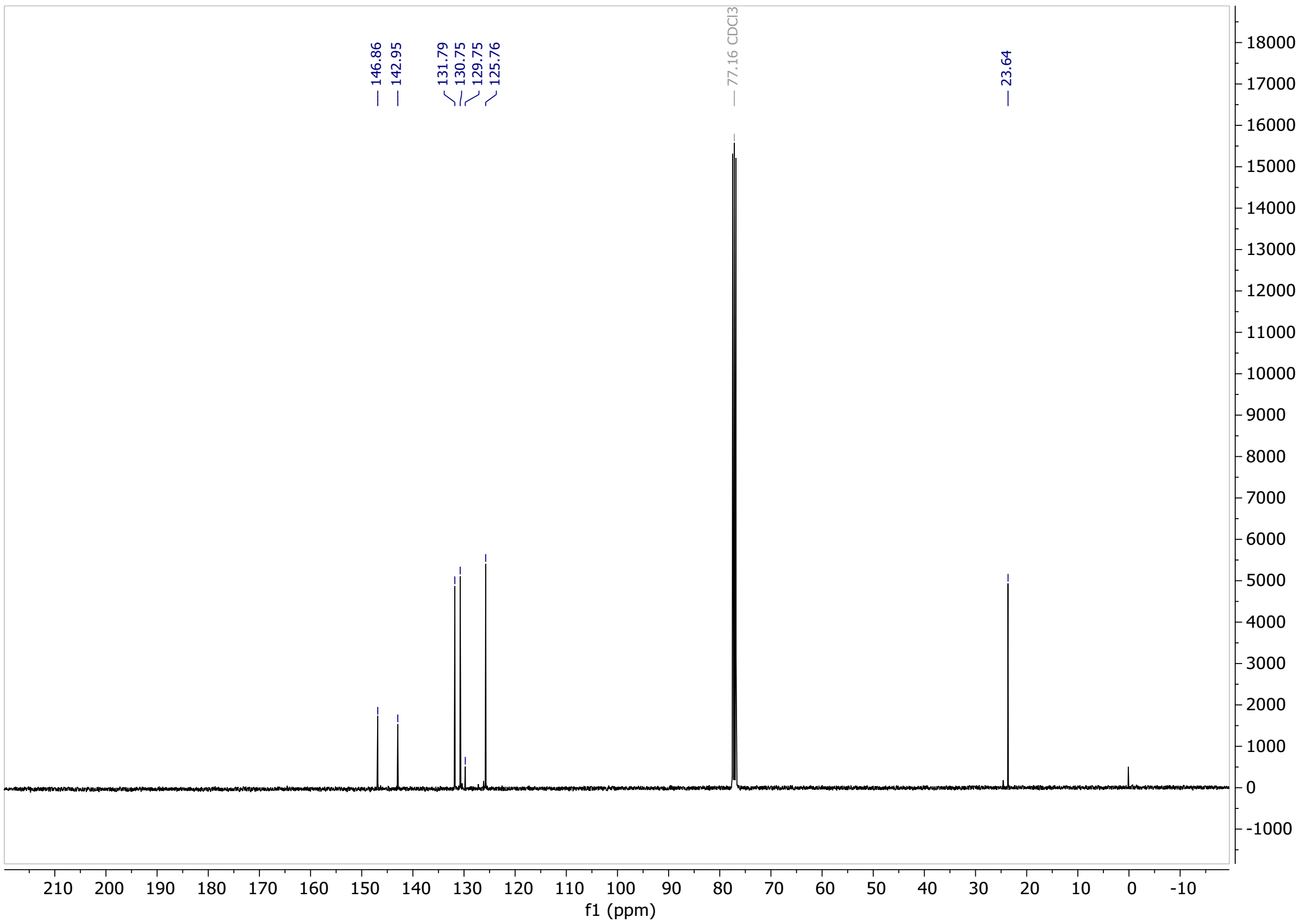

SI Figure S14. ^{1}H- and ^{13}C-spectrum of intermediate **10**.

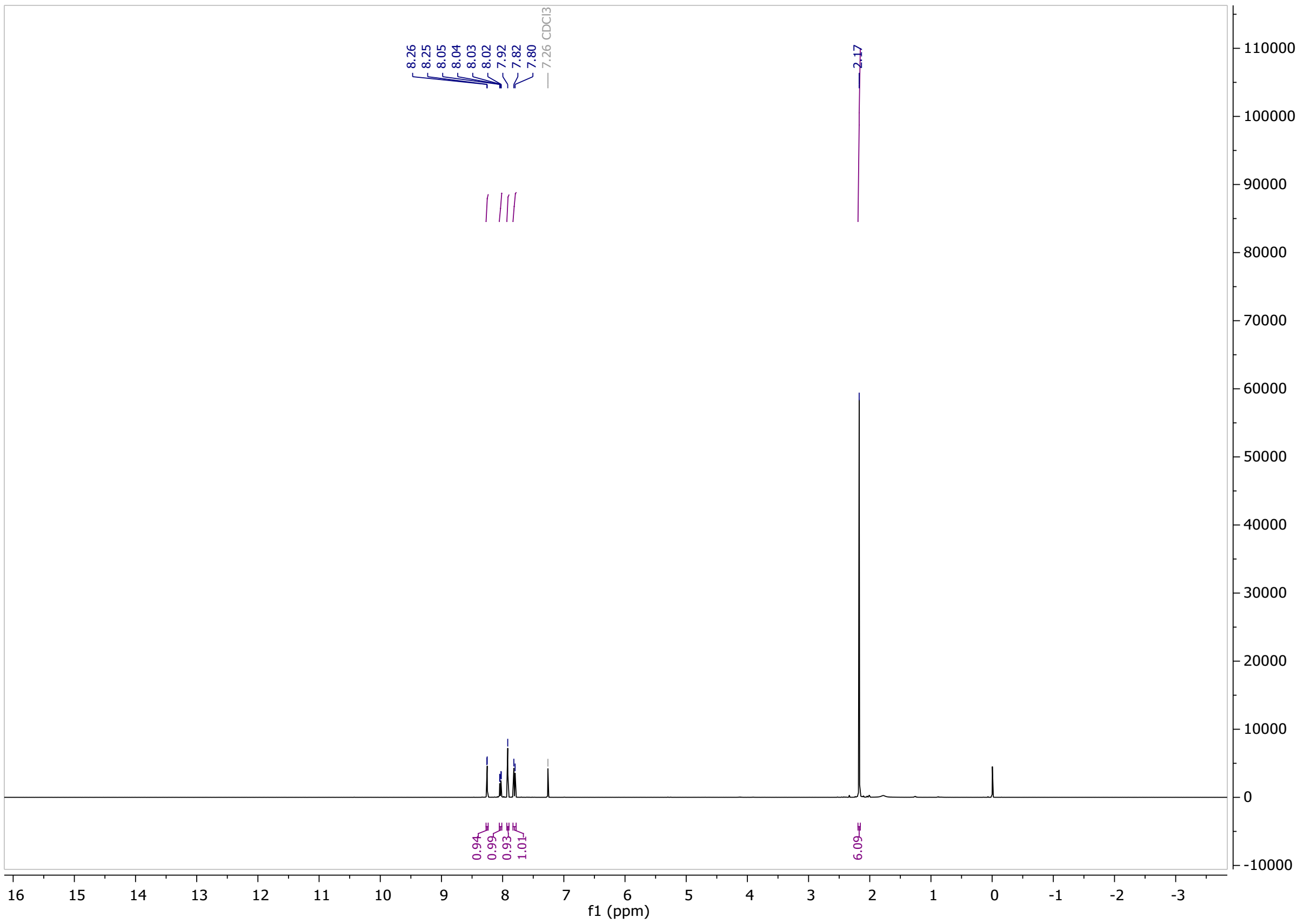

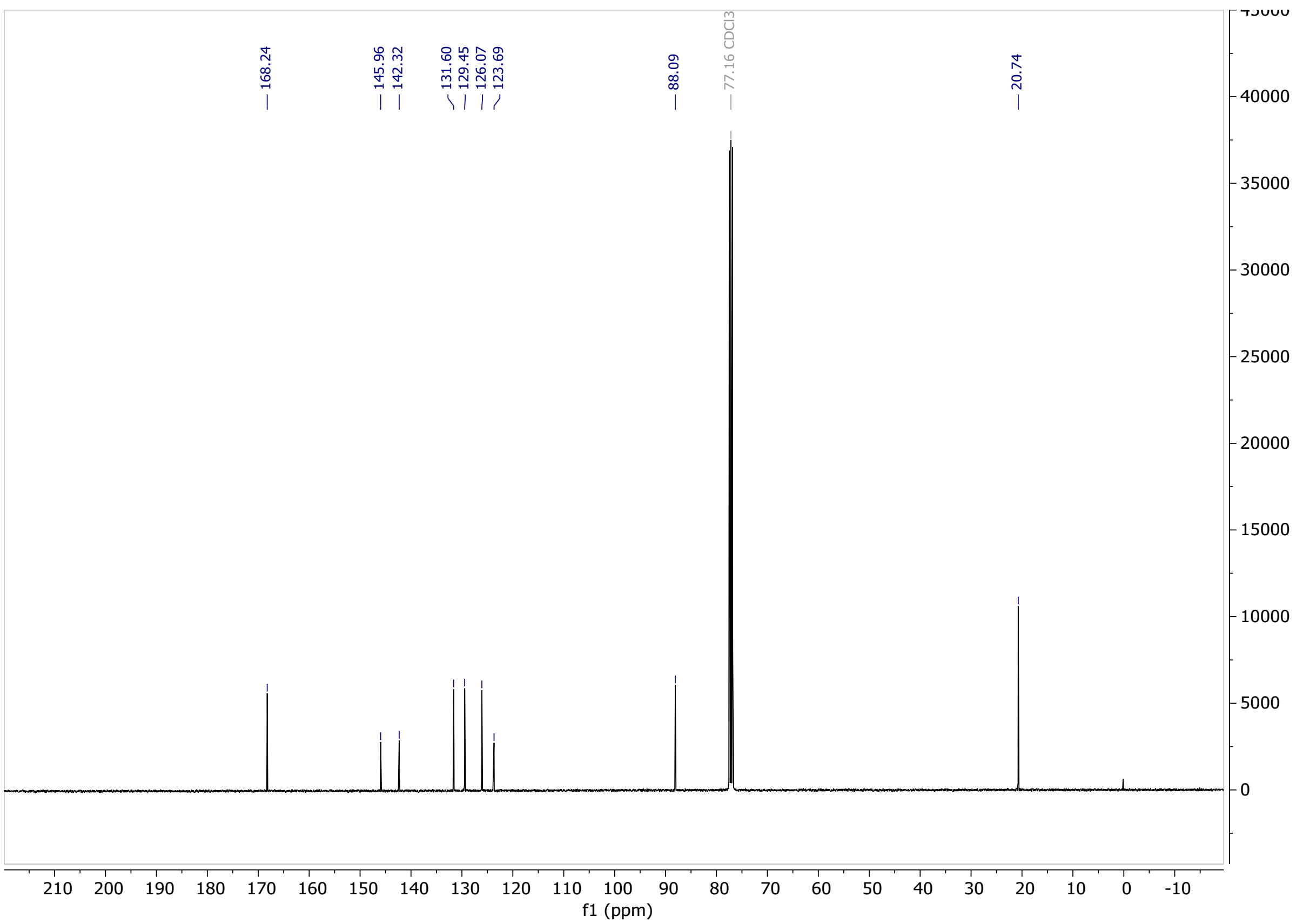

SI Figure S15. ^{1}H- and ^{13}C-spectrum of intermediate/building block **11**.

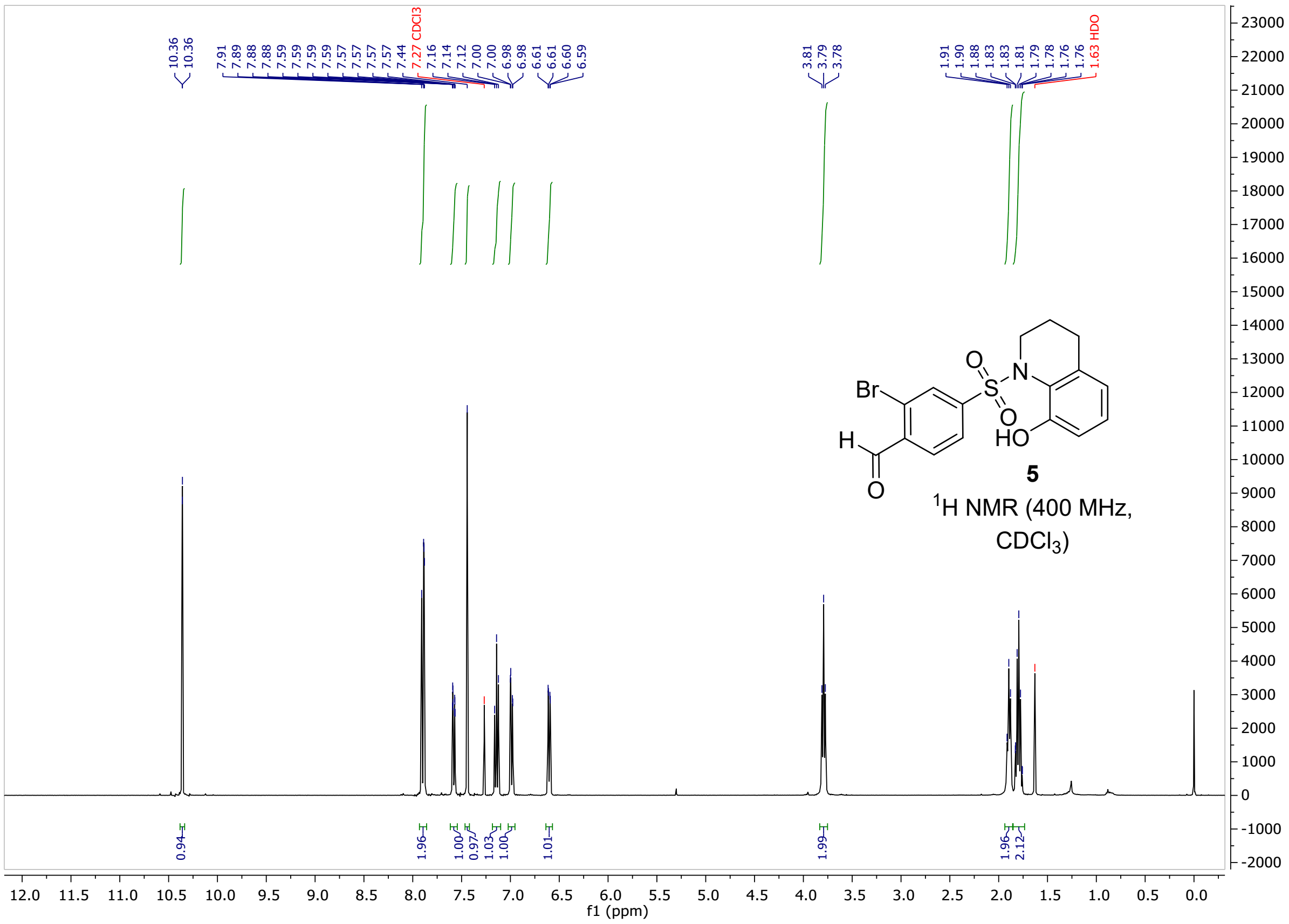

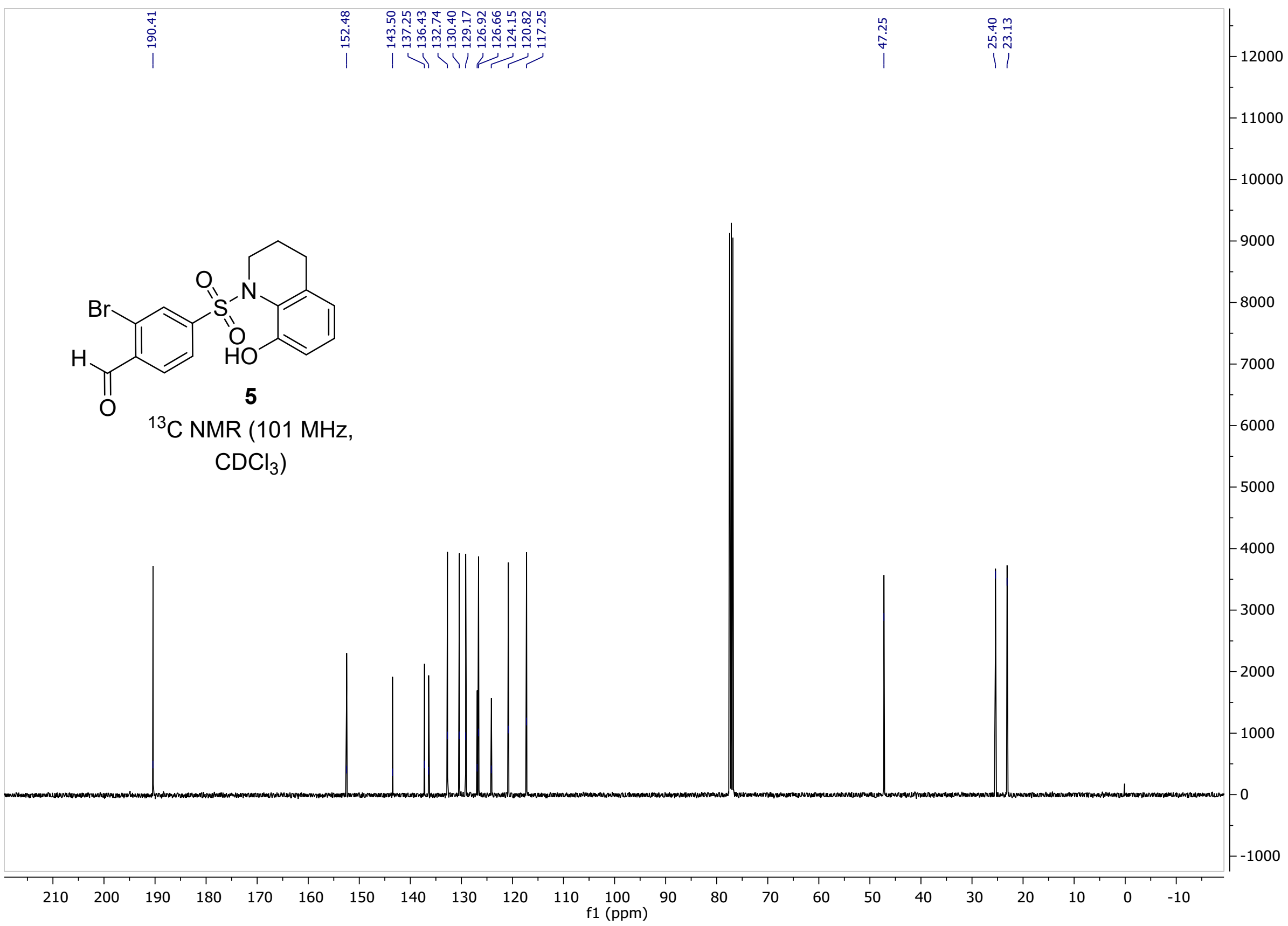

SI Figure S16. ^{1}H- and $^{13}C\{1H\}$-spectrum of aldehyde **5**.

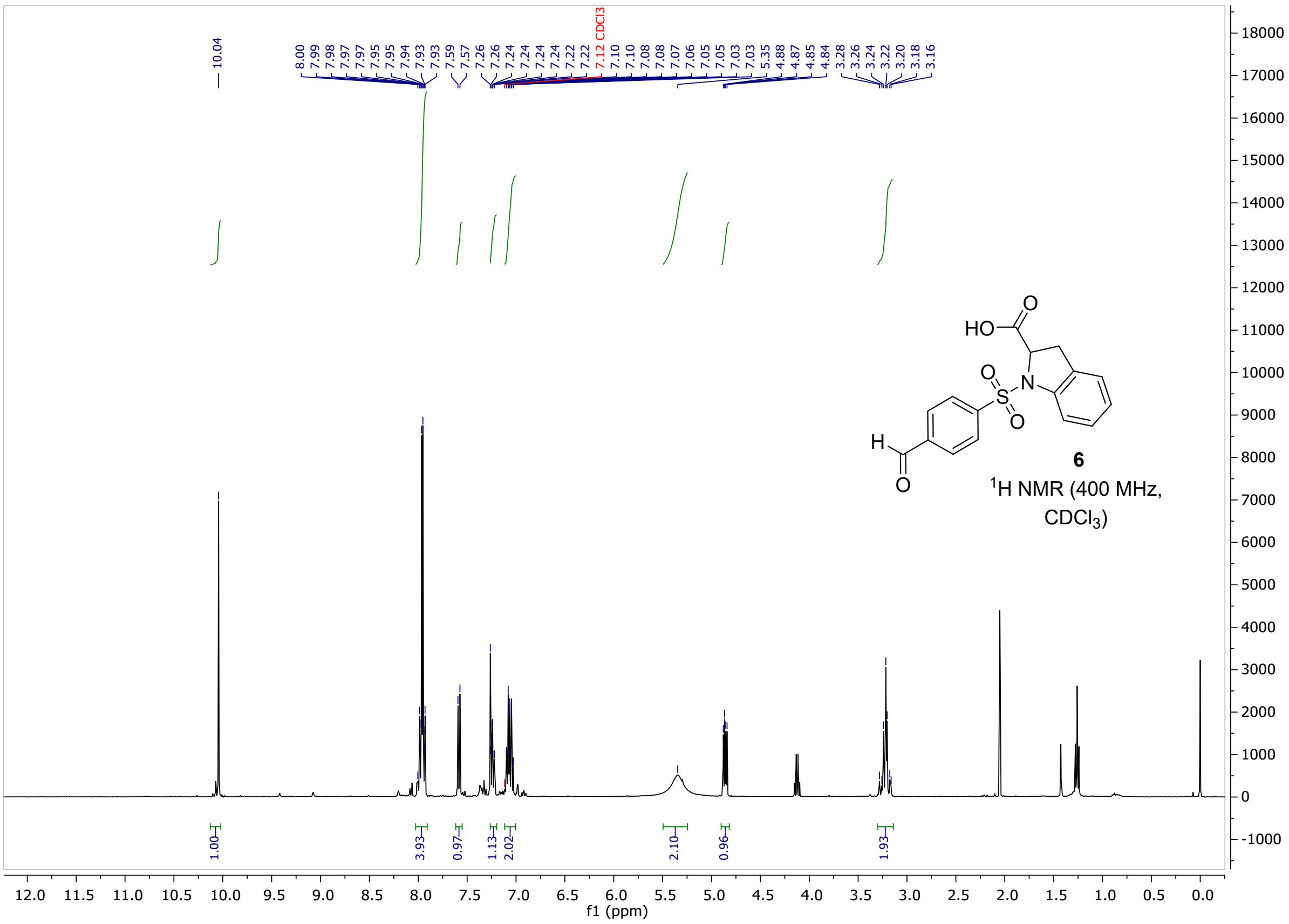

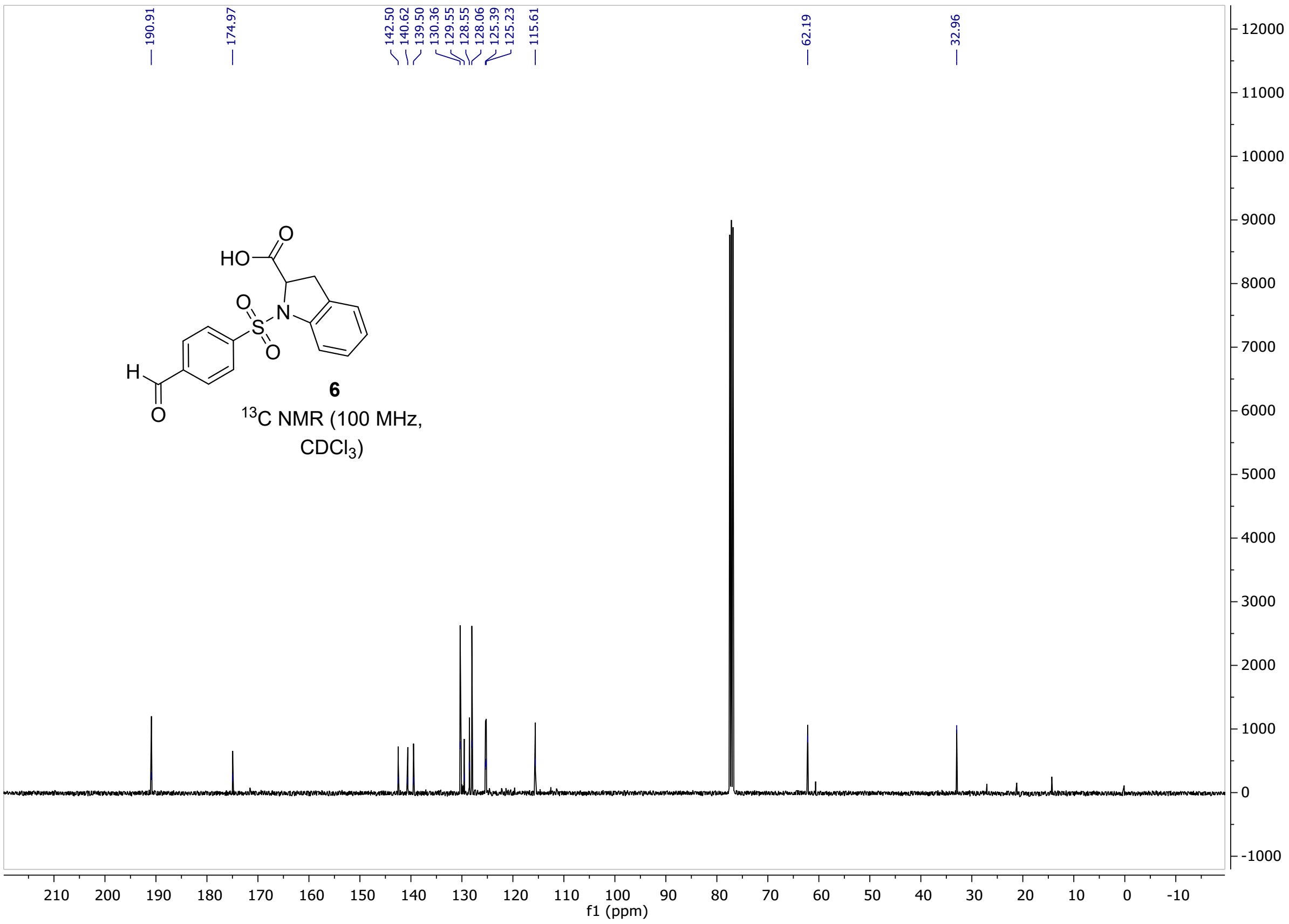

SI Figure S17. ^{1}H- and ^{13}C{1H}-spectrum of aldehyde **6**.

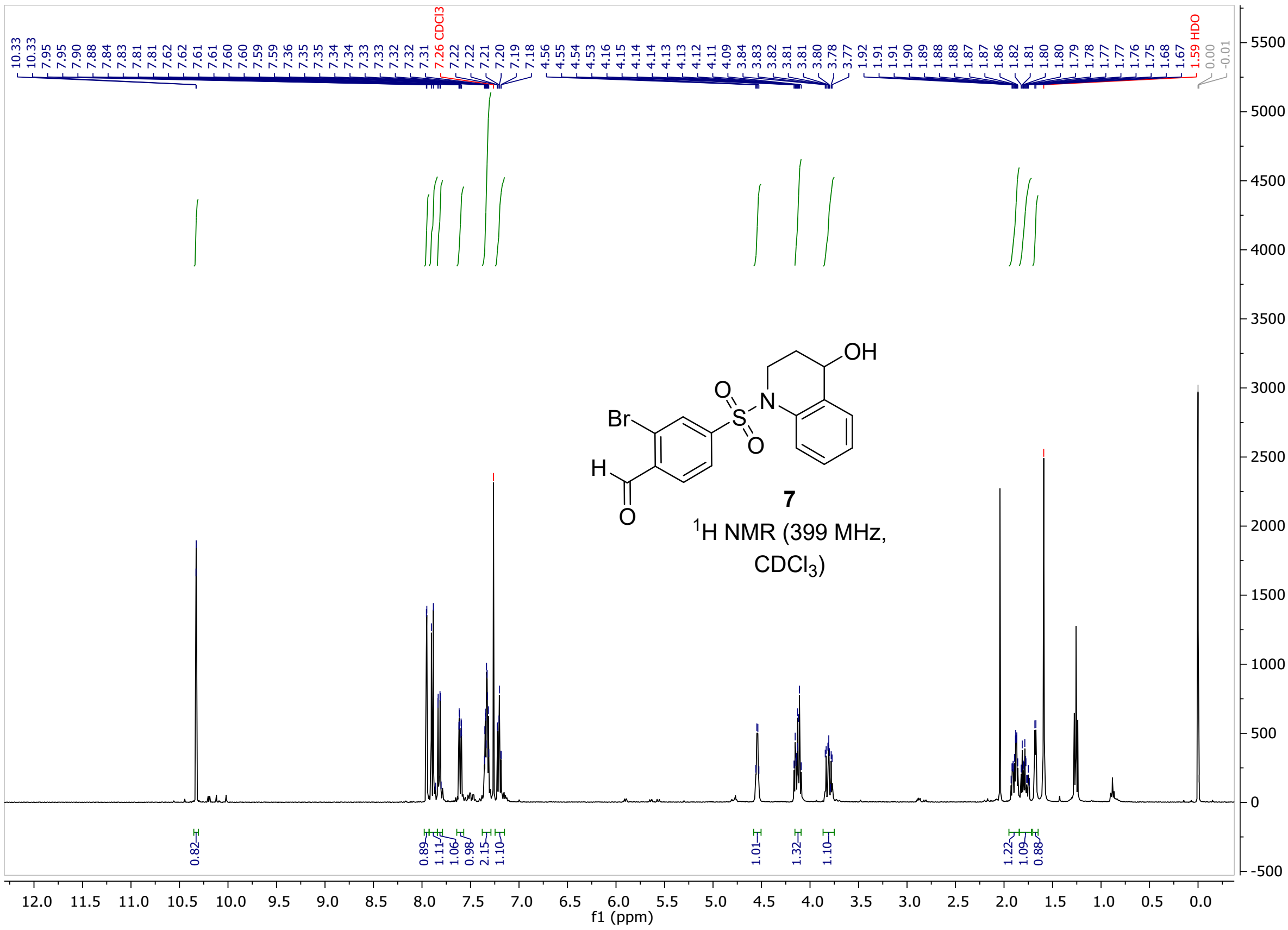
10.33
10.33
7.95
7.95
7.90
7.88
7.84
7.83
7.81
7.81
7.62
7.62
7.61
7.61
7.60
7.59
7.59
7.36
7.35
7.35
7.34
7.34
7.33
7.33
7.33
7.32
7.32
7.31
7.26 CDCl3
7.22
7.22
7.21
7.20
7.19
7.18
4.56
4.55
4.54
4.53
4.16
4.15
4.14
4.14
4.13
4.13
4.12
4.11
4.09
3.84
3.83
3.82
3.81
3.81
3.80
3.78
3.77
1.92
1.91
1.91
1.90
1.89
1.88
1.88
1.87
1.87
1.86
1.82
1.81
1.80
1.80
1.79
1.78
1.77
1.77
1.76
1.75
1.68
1.67
1.59 HDO
0.00
-0.01
OH
O
S
N
O
Br
H
O
7
1H NMR (399 MHz, CDCl3)
0.82
0.89
1.11
1.06
0.98
2.15
1.10
1.01
1.32
1.10
1.22
1.09
0.88
f1 (ppm)

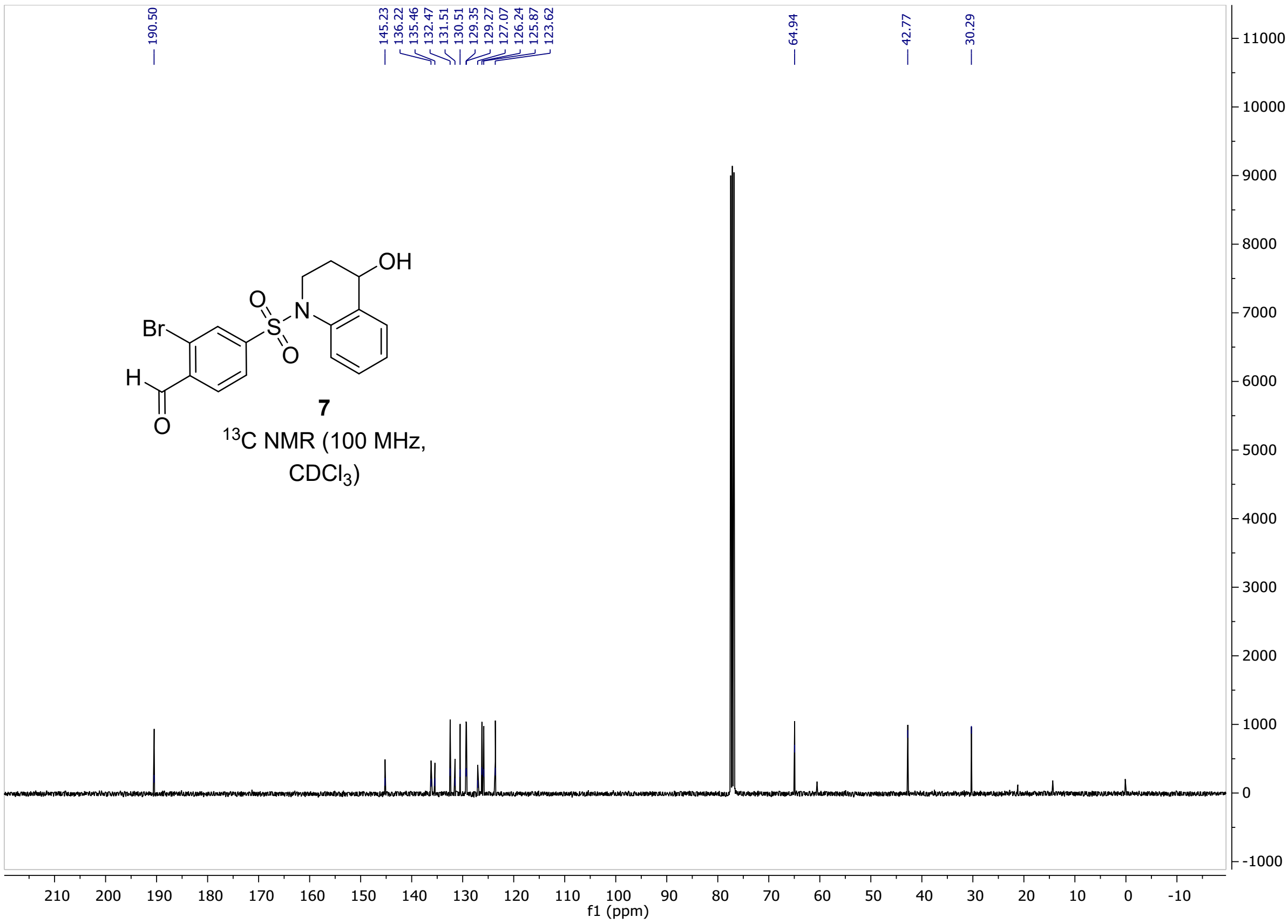
190.50
145.23
136.22
135.46
132.47
131.51
130.51
129.35
129.27
127.07
126.24
125.87
123.62
64.94
42.77
30.29
OH
O
S
N
O
Br
H
O
7
13C NMR (100 MHz, CDCl3)
f1 (ppm)

SI Figure S18. ^{1}H- and ^{13}C{1H}-spectrum of aldehyde **7**.

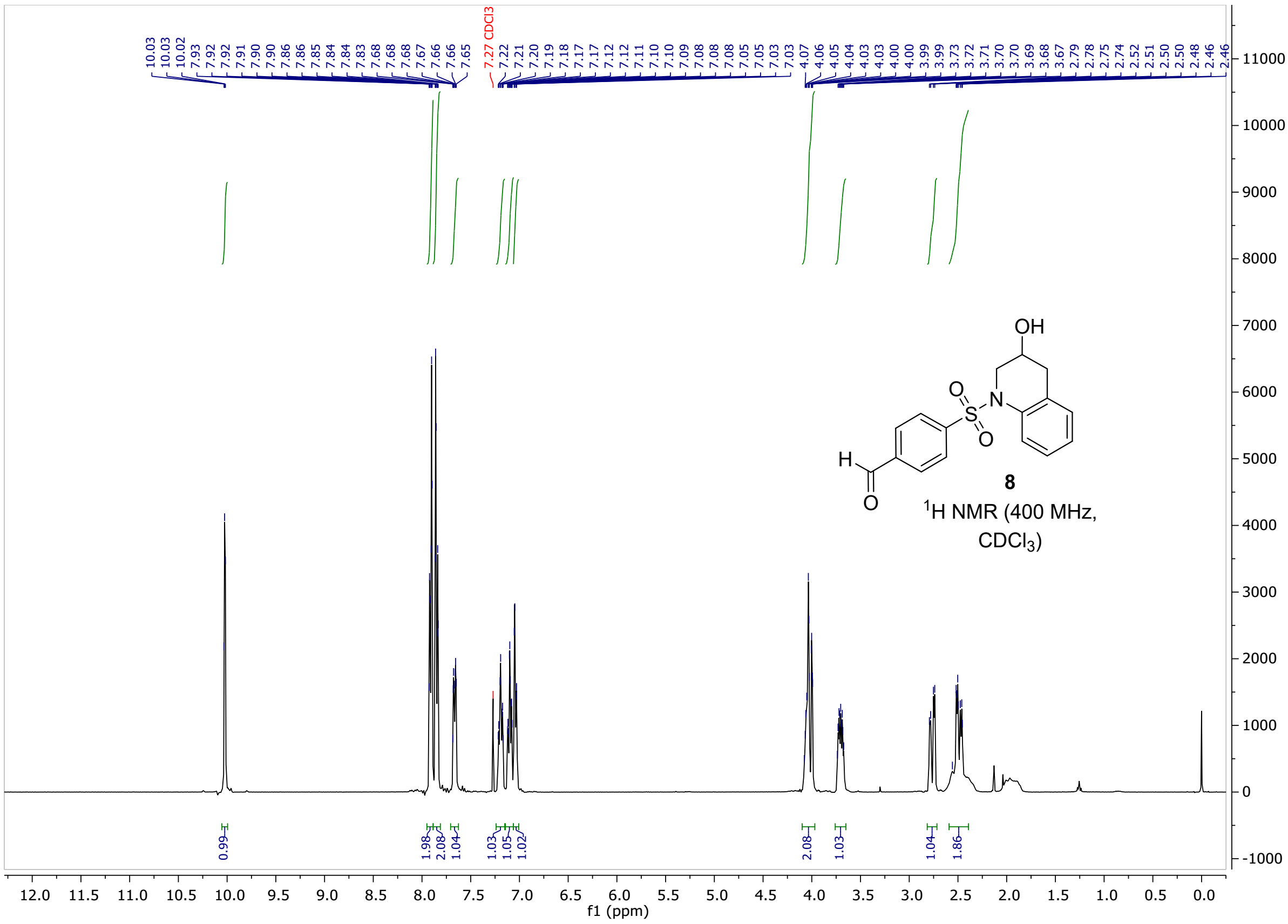

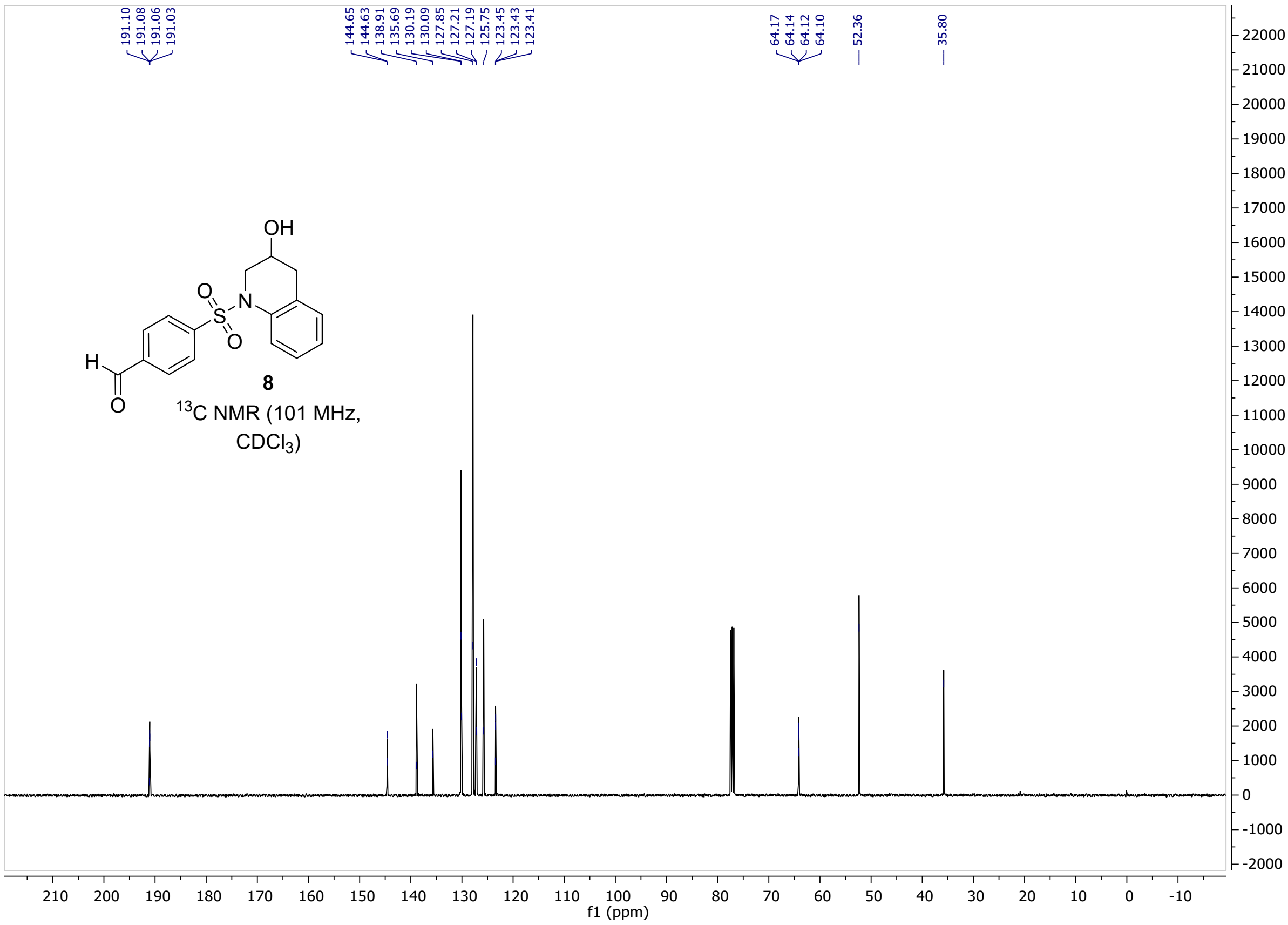

SI Figure S19. ^{1}H- and ^{13}C{1H}-spectrum of aldehyde **8**.

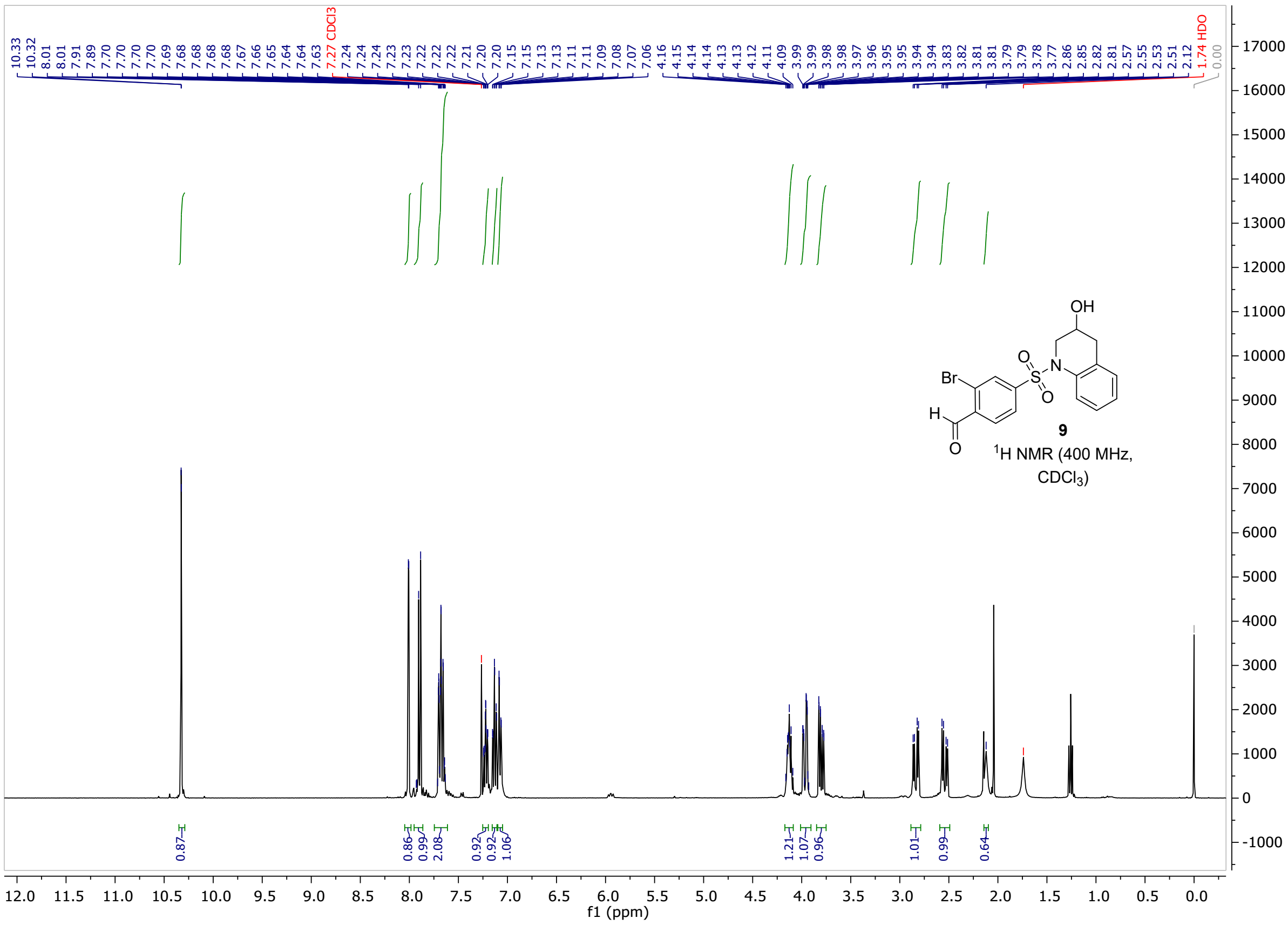

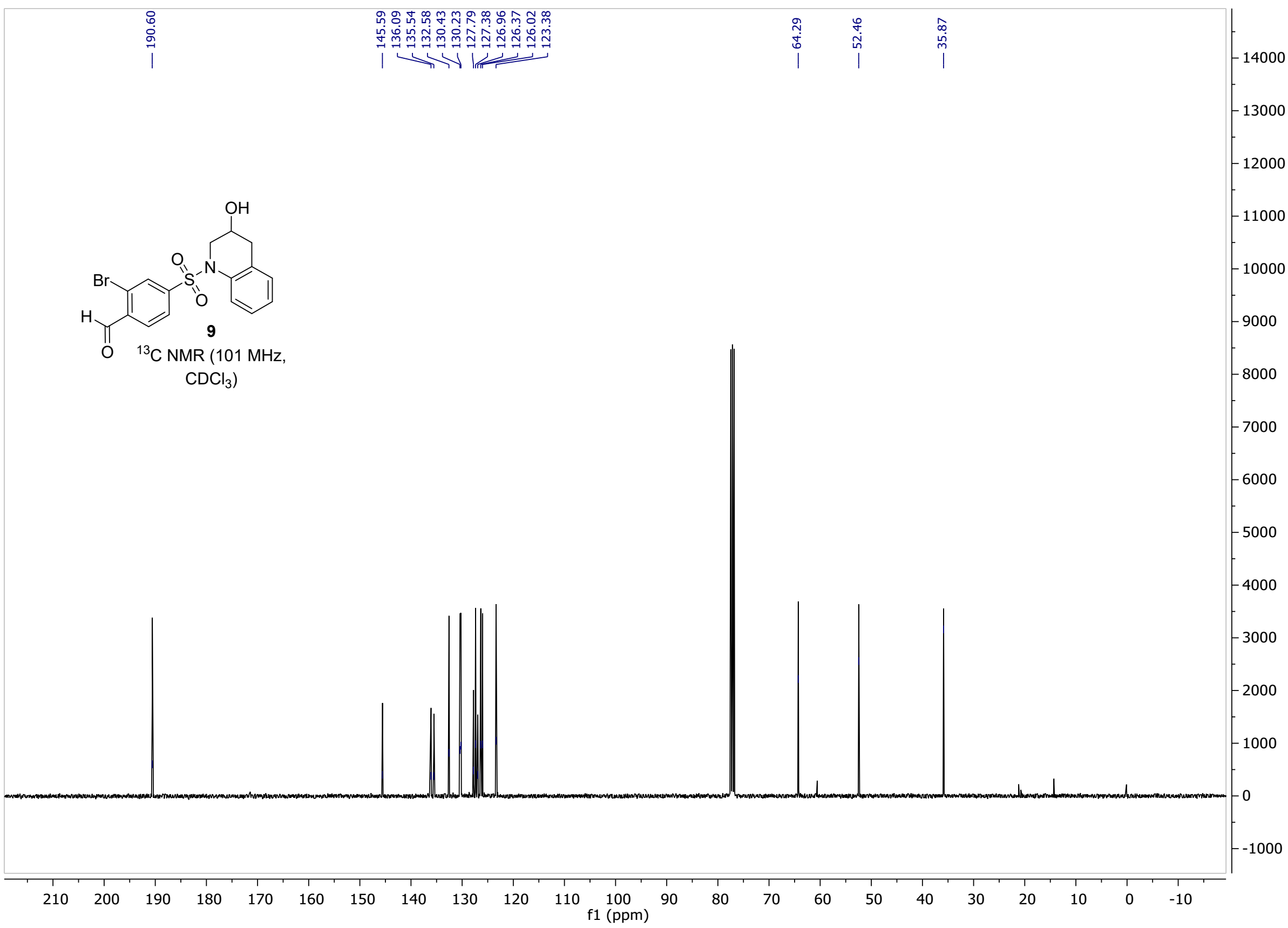

SI Figure S20. ^{1}H- and ^{13}C{1H}-spectrum of aldehyde **9**.

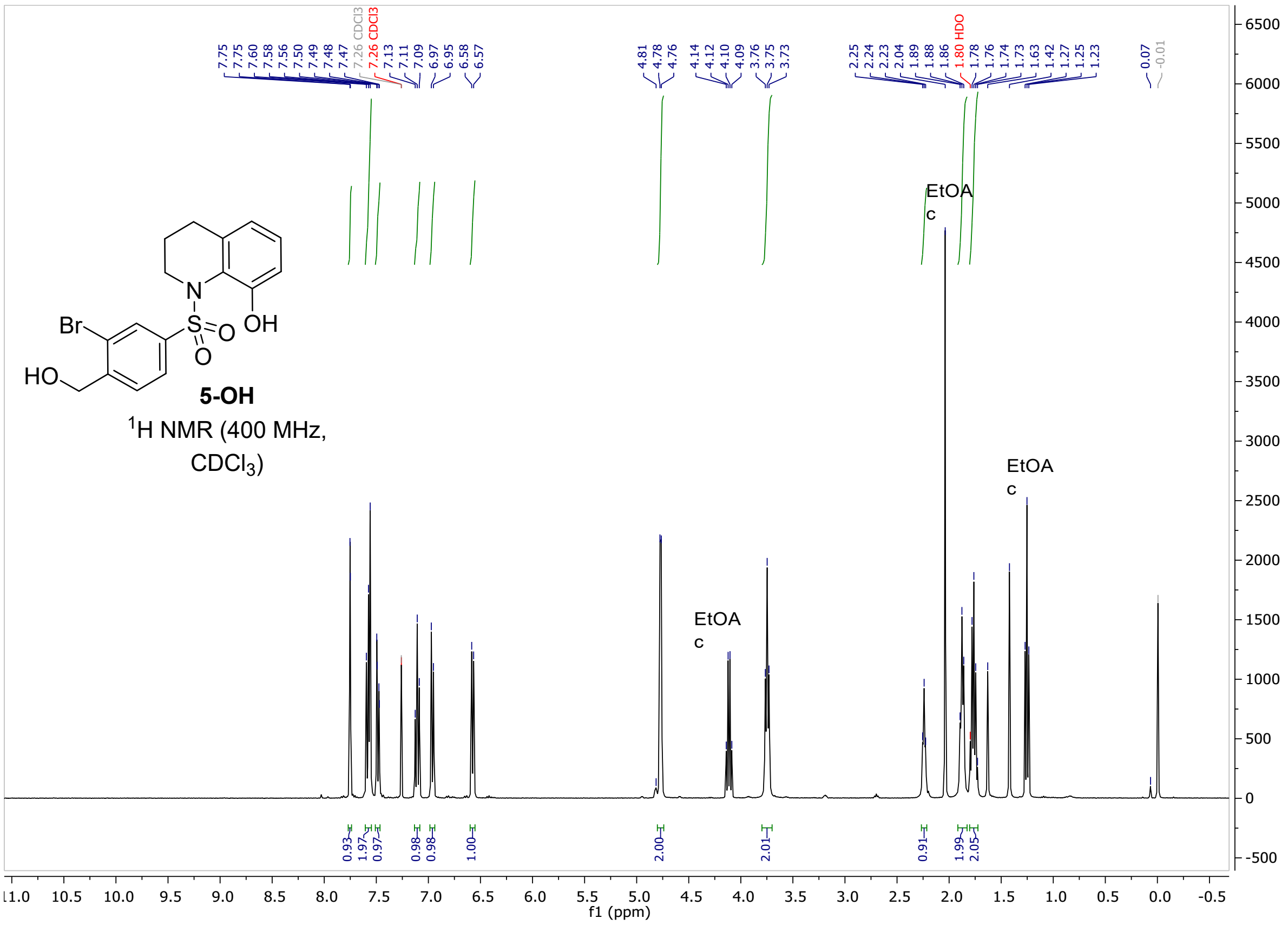

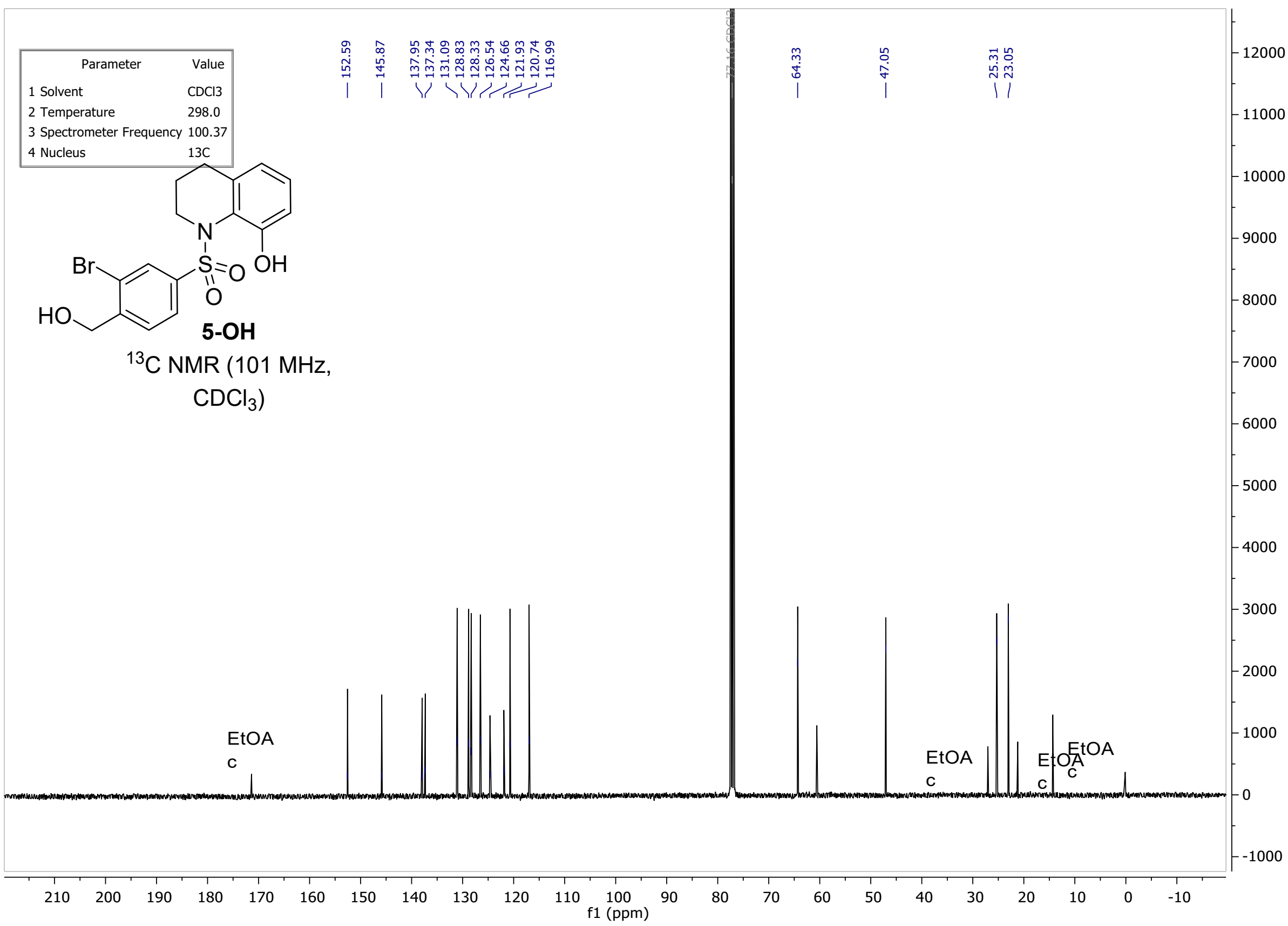

SI Figure S21. ^{1}H- and ^{13}C{1H}-spectrum of alcohol **5-OH**.

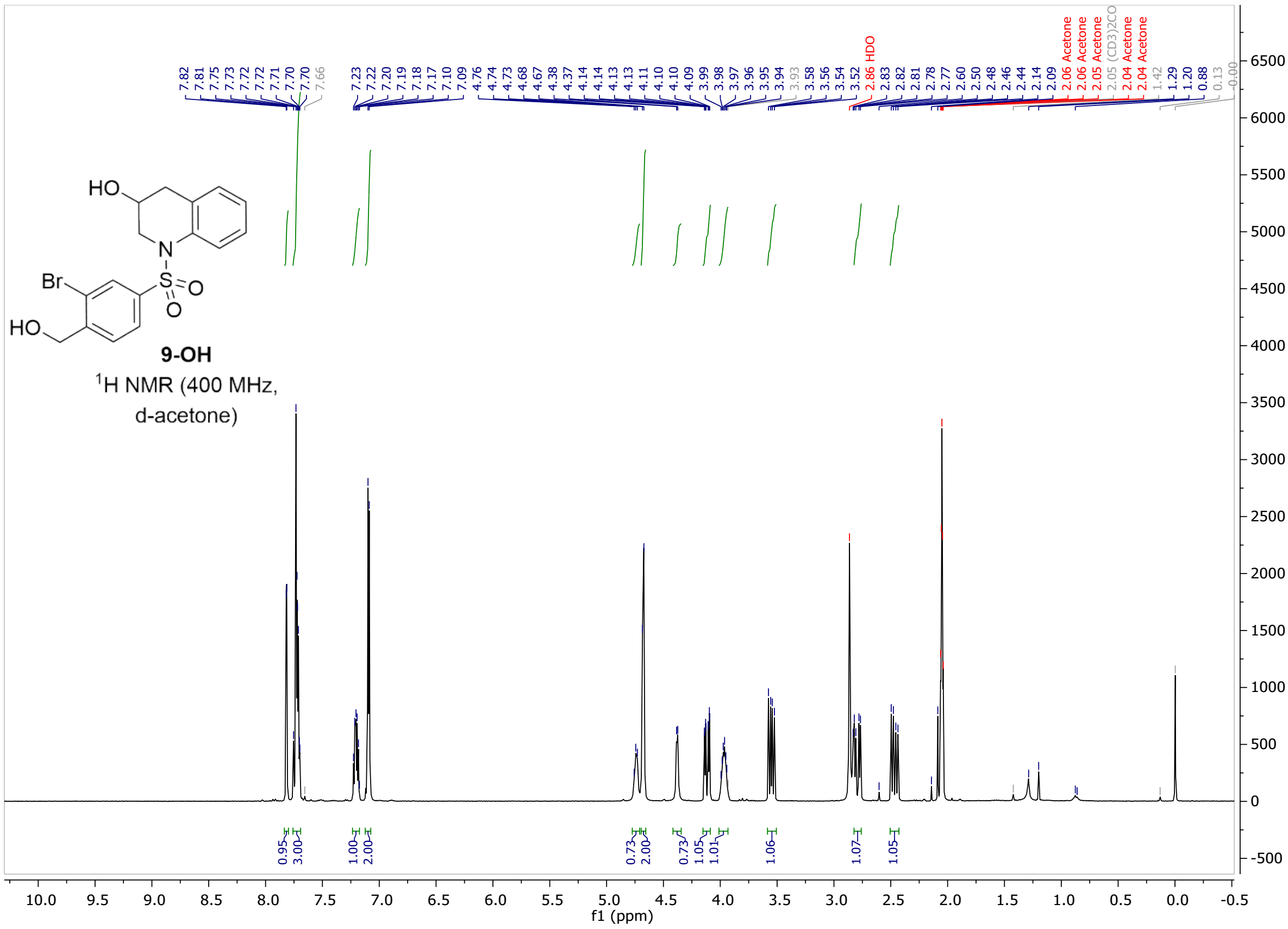

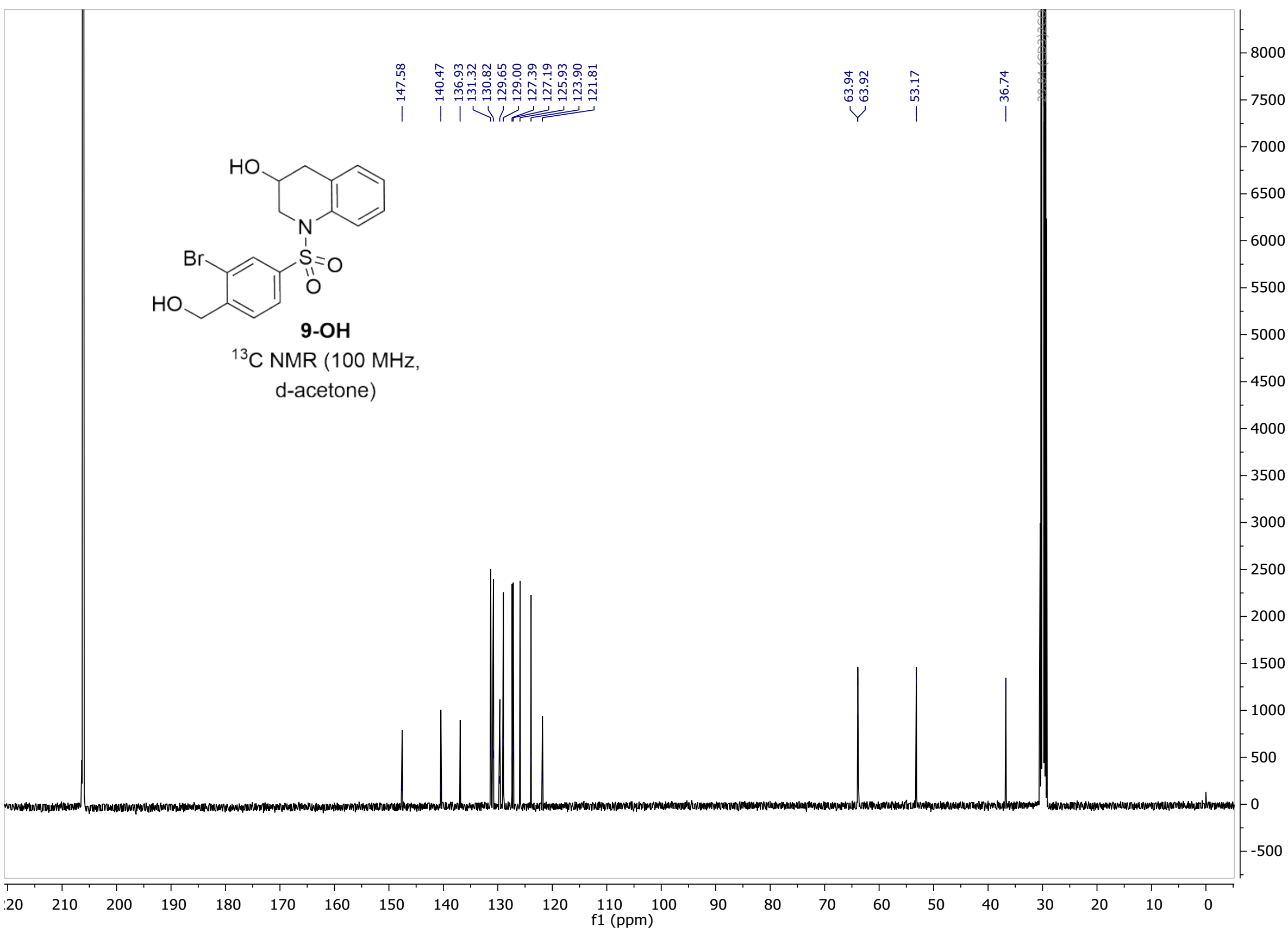

SI Figure S22. ^{1}H- and ^{13}C{1H}-spectrum of alcohol **9-OH**.

Supporting References

[38] S. Barghorn, J. Biernat, E. Mandelkow, *Methods in Molecular Biology* **2005**, *299*, 35–51.

[39] J. Hochmair, C. Exner, M. Franck, A. Dominguez-Baquero, L. Diez, H. Brognaro, M. L. Kraushar, T. Mielke, H. Radbruch, S. Kaniyappan, S. Falke, E. Mandelkow, C. Betzel, S. Wegmann, *EMBO J.* **2022**, *41*, e108882.

[40] J. Hochmair, M. C. M. van den Oetelaar, L. Ravatt, L. Diez, L. J. M. Lemmens, R. Ponce-Lina, R. Sankar, M. Franck, G. Nolte, E. Semenova, S. Mohapatra, C. Ottmann, L. Brunsveld and S. Wegmann, *Commun. Biol.*, **2025**, *8*, 1139.

[41] G. Winter, J. Beilsten-Edmands, N. Devenish, M. Gerstel, R. J. Gildea, D. McDonagh, E. Pascal, D. G. Waterman, B. H. Williams, G. Evans, *Protein Science* **2022**, *31*, 232–250.

[42] L. Potterton, J. Agirre, C. Ballard, K. Cowtan, E. Dodson, P. R. Evans, H. T. Jenkins, R. Keegan, E. Krissinel, K. Stevenson, A. Lebedev, S. J. McNicholas, R. A. Nicholls, M. Noble, N. S. Pannu, C. Roth, G. Sheldrick, P. Skubak, J. Turkenburg, V. Uski, F. Von Delft, D. Waterman, K. Wilson, M. Winn, M. Wojdyr, *Acta Crystallogr. D Struct. Biol.* **2018**, *74*, 68–84.

[43] P. R. Evans, *Acta Crystallogr. D Biol. Crystallogr.* **2011**, *67*, 282–292.

[44] P. R. Evans, G. N. Murshudov, *Acta Crystallogr. D Biol. Crystallogr.* **2013**, *69*, 1204–1214.

[45] A. J. McCoy, R. W. Grosse-Kunstleve, P. D. Adams, M. D. Winn, L. C. Storoni, R. J. Read, *J. Appl. Crystallogr.* **2007**, *40*, 658–674.

[46] G. N. Murshudov, P. Skubák, A. A. Lebedev, N. S. Pannu, R. A. Steiner, R. A. Nicholls, M. D. Winn, F. Long, A. A. Vagin, *Acta Crystallogr. D Biol. Crystallogr.* **2011**, *67*, 355–367.

[47] O. Kovalevskiy, R. A. Nicholls, F. Long, A. Carlon, G. N. Murshudov, *Acta Crystallogr. D Struct. Biol.* **2018**, *74*, 215–227.

[48] P. Emsley, B. Lohkamp, W. G. Scott, K. Cowtan, *Acta Crystallogr. D Biol. Crystallogr.* **2010**, *66*, 486–501.

[49] F. Long, R. A. Nicholls, P. Emsley, S. Gražulis, A. Merkys, A. Vaitkus, G. N. Murshudov, *Acta Crystallogr. D Struct. Biol.* **2017**, *73*, 112–122.

[50] E. Blanc, P. Roversi, C. Vonrhein, C. Flensburg, S. M. Lea, G. Bricogne, *Acta Crystallogr. D Biol. Crystallogr.* **2004**, *60*, 2210–2221.
